# Expanded gene and taxon sampling of diplomonads shows multiple switches to parasitic and free-living lifestyle

**DOI:** 10.1186/s12915-024-02013-w

**Published:** 2024-09-27

**Authors:** Monika M. Wiśniewska, Eric D. Salomaki, Jeffrey D. Silberman, Kristina X. Terpis, Eva Mazancová, Petr Táborský, Vasana Jinatham, Eleni Gentekaki, Ivan Čepička, Martin Kolisko

**Affiliations:** 1 Institute of Parasitology, Biology Centre, Czech Academy of Sciences, Branišovská, Branišovská 1160/31, 2, České Budějovice, 370 05 Czech Republic; 2 Department of Plant Biology and Biotechnology, Faculty of Biotechnology and Horticulture, University of Agriculturein Krakow, 29 Listopada Ave. 54, Kraków, 31-425 Poland; 3 Faculty of Science, University of South Bohemia, Branišovská 1645/31a, České Budějovice, 370 05 Czech Republic; 4 Center for Computational Biology of Human Disease and Center for Computation and Visualization, Brown University, Providence, RI USA; 5 Department of Biological Sciences, University of Arkansas, Fayetteville, AR 72701 USA; 6 Department of Biological Sciences, University of Rhode Island, Kingston, RI 02881 USA; 7 Department of Zoology, Faculty of Science, Charles University, Viničná 7, Prague 2, 128 00 Czech Republic; 8 School of Science, Mae Fah Luang University, 333 Moo1Chiang Rai 57100, Thasud, Muang Thailand; 9 Department of Veterinary Medicine, University of Nicosia Veterinary School, 93 Agiou Nikolaou Street, Nicosia, 2414 Cyprus; 10 Department of Parasitology, Faculty of Science, Charles University, BIOCEV, Vestec 252 42 Czech Republic

**Keywords:** Diplomonads, Phylogenomics, Phylogenetics, Transcriptomics, Parasitic ancestry signals

## Abstract

**Background:**

Diplomonads are anaerobic flagellates classified within Metamonada. They contain both host-associated commensals and parasites that reside in the intestinal tracts of animals, including humans (e.g., *Giardia intestinalis*), as well as free-living representatives that inhabit freshwater and marine anoxic sediments (e.g., *Hexamita inflata*). The evolutionary trajectories within this group are particularly unusual as the free-living taxa appear to be nested within a clade of host-associated species, suggesting a reversal from host-dependence to a secondarily free-living lifestyle. This is thought to be an exceedingly rare event as parasites often lose genes for metabolic pathways that are essential to a free-living life strategy, as they become increasingly reliant on their host for nutrients and metabolites. To revert to a free-living lifestyle would require the reconstruction of numerous metabolic pathways. All previous studies of diplomonad evolution suffered from either low taxon sampling, low gene sampling, or both, especially among free-living diplomonads, which has weakened the phylogenetic resolution and hindered evolutionary insights into this fascinating transition.

**Results:**

We sequenced transcriptomes from 1 host-associated and 13 free-living diplomonad isolates; expanding the genome scale data sampling for diplomonads by roughly threefold. Phylogenomic analyses clearly show that free-living diplomonads form several branches nested within endobiotic species. Moreover, the phylogenetic distribution of genes related to an endobiotic lifestyle suggest their acquisition at the root of diplomonads, while traces of these genes have been identified in free-living diplomonads as well. Based on these results, we propose an evolutionary scenario of ancestral and derived lifestyle transitions across diplomonads.

**Conclusions:**

Free-living taxa form several clades nested within endobiotic taxa in our phylogenomic analyses, implying multiple transitions between free-living and endobiotic lifestyles. The evolutionary history of numerous virulence factors corroborates the inference of an endobiotic ancestry of diplomonads, suggesting that there have been several reversals to a free-living lifestyle. Regaining host independence may have been facilitated by a subset of laterally transferred genes. We conclude that the extant diversity of diplomonads has evolved from a non-specialized endobiont, with some taxa becoming highly specialized parasites, others becoming free-living, and some becoming capable of both free-living and endobiotic lifestyles.

**Supplementary Information:**

The online version contains supplementary material available at 10.1186/s12915-024-02013-w.

## Highlights

• Expanding the taxon sampling of phylogenomic analyses for diplomonads by sequencing transcriptomes of 13 free-living isolates and one endobiotic isolate.

• Constructing a well-resolved evolutionary framework for exploring lifestyle adaptation in diplomonads using a phylogenomic approach.

• Identifying genetic signatures of parasitic ancestry (variant-specific surface proteins, virulence factors) in free-living diplomonads.

**Supplementary Information:**

The online version contains supplementary material available at 10.1186/s12915-024-02013-w.

## Background

Diplomonads are a group of anaerobic, commonly endobiotic, flagellates that belong to the group Metamonada. *Giardia intestinalis* and *Spironucleus salmonicida* are the most widely known diplomonads, as they are the causative agents of diarrheal disease in humans and other mammals and systemic infections in salmonid fish, respectively [1–4]. Diplomonads also contain several known, but under-studied free-living species, for example members of the genera *Hexamita* and *Trepomonas* [5–8]. Diplomonads are divided into two major groups: Hexamitinae and Giardiinae. All known members of Giardiinae are endobionts while Hexamitinae contains both endobionts and free-living organisms. Typical diplomonads possess two sets of nuclei and flagella with their associated cytoskeleton, giving the impression of “doubled” cells, and were therefore named Diplomonadida. However, there are also enteromonads, which belong to Hexamitinae and possess only a single nucleus and one set of flagella with the associated cytoskeleton. Previous studies have shown that these two morphologies are interspersed in phylogenies, suggesting that the switch from one to the other was not uncommon during diplomonad evolutionary history [6]. All known members of the Hexamitinae utilize an alternative genetic code, in which two of the canonical termination codons (TAA and TAG) encode glutamine, leaving TGA as the only stop codon [6, 9, 10]. Diplomonads also lack classical mitochondria and instead retain highly reduced anaerobic mitochondrial related organelles [11–13]. Their importance as parasites of humans and livestock, as well as their unique morphologies and reduced mitochondria made diplomonads early targets for cellular and evolutionary investigations, including genome sequencing [14–16]. Indeed, several high-quality genomes of *Giardia* have been published, as well as that of *Spironucleus salmonicida* [4, 14, 15].

Parasitism is a remarkably common life strategy that has evolved numerous times across the eukaryotic tree of life. For example, the Tara Oceans project reported that 59% of small subunit rRNA gene (SSU rRNA) richness belonged to parasitic protists [17]. Genomic modifications often occur during parasite evolution facilitating their survival and adaptation to a host-associated habitat, as has been observed in the genomes of *Giardia* and *Spironucleus* [4, 15, 18–20]. Novel genes and gene families often arise through gene duplications and massive gene family expansions that enable parasites to transport or scavenge essential metabolites from the host and to evade or counteract host immune responses [21–23]. For instance, *G. intestinalis* and *S. salmonicida* encode variant-specific surface proteins (VSPs), which are variably expressed through cell generations and are presumed to be an antigenic adaptation enabling evasion of the host immune system [4, 24]. Additionally, *Giardia* spp. possess components such as pyridoxamine 5’-phosphate oxidase (PNPO), extracellular nuclease, cathepsin B, and tenascins that are believed to facilitate successful colonization of the host intestine [25]. Along with genetic expansion, parasite genomes are often streamlined, as they no longer require certain pathways for producing metabolites that they acquire from host environments [26–29]. Due to the difficulty of regaining previously lost essential metabolic capacities, it is thought that organisms transitioning from a parasitic ancestry back to a free-living lifestyle are exceedingly rare [21, 29–33]. Diplomonads seemingly represent one of the few documented lineages in which such a transition may have occurred [21, 29–31]. Previously, single to few loci phylogenetic analyses have supported the hypothesis that several free-living diplomonad species are nested within a clade of host-associated species, suggesting a reversal from parasitism or host reliance to a free-living lifestyle [34, 35]. Recent studies have shown a greater diversity of free-living diplomonads than was previously recognized [8, 34, 35].

Xu et al. [30] sequenced the transcriptome of *Trepomonas* sp. isolate PC1 to explore the metabolic capacity of a free-living diplomonad. When compared to *Giardia* and *Spironucleus*, *Trepomonas* sp. PC1 showed an increase in its metabolic capabilities, notably driven by additional genes involved in carbohydrate degradation and nucleotide metabolism pathways, which the authors attributed to adaptation to a more variable habitat. To explore the origin of this increased coding capacity, phylogenetic analyses of all *Trepomonas* sp. PC1 transcripts were performed, which identified 271 putative horizontal gene transfer (HGT) events [30]. Many of the described HGTs were involved in bacterivory and the utilization of metabolic products from their prey. However, this single transcriptome represents the only genome-scale data available for free-living diplomonads to date.

Here, we address previous shortcomings in our understanding of free-living diplomonad evolution by generating transcriptomes from thirteen free-living and one endobiotic diplomonad. We used these data to perform phylogenomic analyses and to identify potential indicators of parasitic ancestry in the free-living species. Our phylogenomic analysis of Metamonada species placed these taxa as a crown lineage, having arisen from a common ancestor shared with several parasitic lineages, including members of the genera *Spironucleus* and *Giardia*. These results provide robust support for lineages of free-living diplomonads being nested within endobiotic species that retain molecular evidence of parasitic ancestry.

## Results

**Fig. 1:**
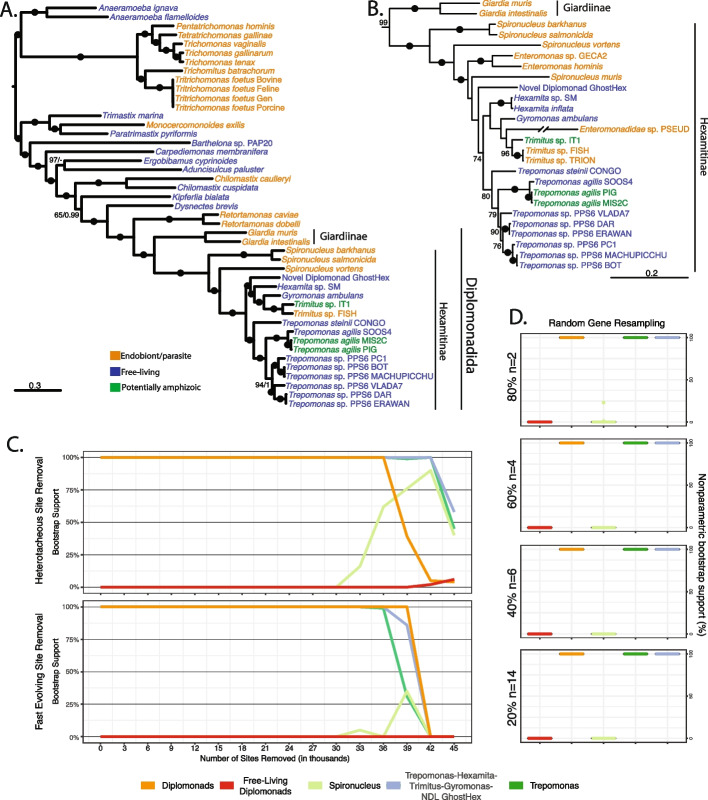
**A** Maximum likelihood tree of diplomonads and other members of Metamonada inferred from 188 genes in IQ-TREE using C60 + LG + G + F model and 1000 PMSF bootstraps. The tree was rooted based on Stairs et al. 2021. Statistical support on branches are Bootstrap support (BP)/Posterior Probability (PP). Black dots denote 100% BP support and 1 PP support. Support lower than 70% BP and PP < 0.9 not shown. **B** Maximum likelihood tree of diplomonads and other members of Metamonada constructed based on 4 genes in IQ-TREE with C60 + LG + G + F and GTR + G models for amino acid and SSU rRNA sequences respectively. Statistical support on branches is BP support. Black dots denote 100% BP support and values under 70% BP are not shown. **C** Support for specific groups of interest along a series of datasets made by serial removal of fast evolving and heterotacheous sites. **D** Support for groups of interest in random gene resampling datasets. Each boxplot shows the spread of support values in different gene samplings, here the support is almost always the same

We generated transcriptomes from 14 diplomonads: 9 *Trepomonas* spp., 1 *Gyromonas ambulans*, 2 *Trimitus* spp., 1 *Hexamita* sp., and 1 novel diplomonad lineage; all but one were isolated from free-living environments. Using 188 genes, we placed these 14 newly sequenced diplomonad isolates (see Additional file 1: Table S1) in the broader context of metamonad evolutionary history (Fig. 1A). When rooted as in Stairs et al. [36], the overall branching pattern of metamonads reflects their recently published topology, where Parabasalia and Anaeramoebae branch sister to each other with maximum support, while Preaxostyla and Fornicata form a fully supported clade. Within the Fornicata, diplomonads received full support in both maximum likelihood (ML) and Bayesian inference (BI) analyses. The earliest diverging diplomonad lineages are comprised exclusively of endobionts (e.g., *Giardia* spp. and *Spironucleus* spp.), with some of them being known pathogens (e.g., *Giardia intestinalis*, *Spironucleus salmonicida*). Our analyses recovered two sister clades that contain predominantly free-living taxa nested deep within the diplomonads, sharing common ancestry with endobionts and parasites. One of these clades is comprised entirely of *Trepomonas* spp., while the other contains the genera *Gyromonas*, *Hexamita*, *Trimitus*, and Hexamitinae sp. GhostHex (novel diplomonad lineage, NDL GhostHex). Analyses of a four-gene dataset with additional endobiotic lineages shows a similar topology and pattern of lifestyle distribution as the phylogenomic tree, with the exception of the weakly supported position of Enteromonad isolate-PSEUD (Fig. 1B).

To further investigate the stability of diplomonad relationships, secondary analyses were conducted on the phylogenomic dataset, including random resampling of genes, and serial removal of heterotacheous (sites with the largest disparity in rates of evolutionary change between slow and fast evolving lineages) and fast evolving sites. Support for monophyly of diplomonads, *Trepomonas* spp., and the *Trepomonas*-*Hexamita*-*Trimitus*-*Gyromonas*-NDL GhostHex clade was unwavering in all secondary analyses (until the entire topology collapsed due to an insufficient amount of data; Fig. 1C, D). In contrast, monophyly of free-living diplomonads was never supported in any analysis, due to the placement of the endobiotic *Trimitus* spp. within the clade that also contains *Hexamita* and *Gyromonas*. Furthermore, an approximately unbiased (AU) test rejected the monophyly of free-living diplomonads.

### Variant-specific surface and other cysteine-rich proteins

We found evidence of VSP presence (Table 1), but also a reduction in the number of these proteins compared to parasitic relatives, and their loss throughout the diplomonad tree. To be considered a VSP-like protein, the sequence must be comprised of > 10% cysteine, have > 5 CXC or CXXC motifs, contain a transmembrane (TM) domain with less than 100 amino acids following the TM, and have an N-terminal conserved motif [4, 15, 18–20]. Proteins with similar characteristics as VSPs that lacked the N-terminal conserved motif were denoted as cysteine-rich membrane proteins (CRMP). Cysteine-rich proteins with a signal peptide but lacking a transmembrane domain were classified as cysteine-rich secreted proteins (CRSP). A relatively small number of identified VSPs and CRMPs possessed a signal peptide, which is unsurprising as diplomonads are known to possess non-typical signal peptides that are difficult to identify [4].

**Table 1:** Number of VSPs, CRMPs and CRSPs identified in each investigated species. Number in brackets shows number of sequences with identifiable signal peptide

	**VSP**	**CRMP**	**CRSP**
*Barthelona* PAP20	**0**	**12 (2)**	**1**
*Carpediemonas membranifera*	**0**	**7**	**4**
*Ergobibamus cyprinoides*	**0**	**3**	**1**
*Aduncisulcus paluster*	**0**	**18**	**1**
*Chilomastix caulleryi*	**0**	**12(1)**	**3**
*Chilomastix cuspidata*	**0**	**2**	**2**
*Kipferlia bialata*	**0**	**4**	**5**
*Dysnectes brevis*	**1**	**58(3)**	**7**
*Retortamonas* cf* caviae*	**1**	**29(1)**	**3**
*Retortamonas dobelli*	**1**	**17(1)**	**4**
*Giardia muris*	**43 (14)**	**25(3)**	**3**
*Giardia intestinalis* genome	**106 (56)**	**85(63)**	**15**
*Giardia intestinalis* transcriptome	**92 (20)**	**67(22)**	**65**
*Spironucleus salmonicida* genome	** 111**	**170(2)**	**1**
*Spironucleus salmonicida* transcriptome	**52 (2)**	**127(6)**	**4**
*Spironucleus barkhanus*	**0**	**0**	**1**
*Spironucleus vortens*	**1**	**24(1)**	**8**
Novel Diplomonad lineage GhostHex	**1**	**73(4)**	**7**
*Hexamita* sp. SM	**28**	**221(12)**	**28**
*Gyromonas ambulans*	**5**	**65(4)**	**22**
*Trimitus* sp. IT1	**2 (1)**	**42(6)**	**12**
*Trimitus* sp. FISH	**2 (1)**	**85(1)**	**4**
*Trepomonas steinii* CONGO	**1**	**45**	**12**
*Trepomonas agilis* SOOS4	**0**	**18(1)**	**1**
*Trepomonas agilis* MIS2C	**0**	**26(1)**	**0**
*Trepomonas agilis* PIG	**1**	**75**	**1**
*Trepomonas* sp PC1	**0**	**20(1)**	**0**
*Trepomonas* PPS6 BOT	**1**	**48(4)**	**1**
*Trepomonas* PPS6 MACHUPICCHU	**1**	**72(8)**	**9**
*Trepomonas* PPS6 VLADA7	**3(1)**	**106(9)**	**56**
*Trepomonas* PPS6 DAR	**0**	**72(6)**	**23**
*Trepomonas* PPS6 ERAWAN	**1**	**42(2)**	**5**

**Fig. 2:**
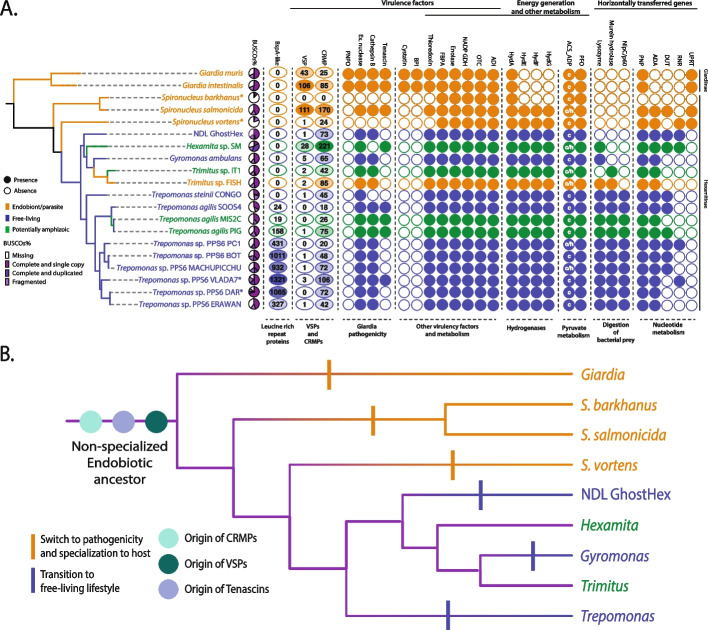
**A** Cartoon phylogenetic tree of diplomonads with mapped presence/absence of BspA-like proteins, putative virulence factors (it is important to note that many enzymes identified as virulence factors might not be truly involved in pathogenesis), genes involved in energy production and other metabolism, and HGTs. Where appropriate, the number of identified copies of each gene is shown. Pie charts represent completeness of the transcriptomes calculated based on the BUSCO (Benchmarking Universal Single-Copy Orthology) scores against eukaryotic lineages shared between diplomonads lineages (See Additional file 5). White letters c and h in ACS_ADP gene marks the presence of putative cytosolic and hydrogenosomal copies. An asterix indicates either incomplete or heavily contaminated dataset. The color of a taxa corresponds to its lifestyle. **B** Hypothetical scenario of lifestyle evolution across diplomonads. Based on the retention pattern of the parasitic traits in the new diplomonad isolates, the last common ancestor of diplomonads was likely endobiotic, but non-host specialized. Some lineages like *Giardia* and *Spironucleus* evolved to be host-specialized pathogens (denoted by orange hash-marks on branches), whereas other lineages like NDL GhostHex, *Gyromonas*, and *Trepomonas* transitioned back to a secondarily free-living lifestyle (denoted by blue hash-marks on branches). Purple branches on the tree indicate hypothesized ancestral state

Pathogenic diplomonads retain a relatively large number of putative VSPs, ranging from 43 (and 25 CRMPs) candidate sequences identified in the *Giardia muris* genome, 106 (and 85 CRMPs) in the *Giardia intestinalis* genome, to 111 (and 170 CRMPs) in the *Spironucleus salmonicida* genome. Only one putative VSP (and 24 CRMPs) sequence was identified in the *Spironucleus vortens* data; however, those data are known to be highly fragmented and incomplete, as are the expressed sequence tag data from *S. barkhanus*, where no VSPs, CRMPs, or CRSPs were identified. A wide range in number of VSP-like sequences were identified in other diplomonads. In the *Hexamita* sp. SM transcriptome, 28 VSP-like sequences (and 221 CRMPs) were found, whereas less than ten VSP-like sequences were found in the *Trepomonas* spp., NDL GhostHex, and *Trimitus* sp. FISH transcriptomes (Fig. 2A). Most Hexamitinae diplomonads appear to use the same conserved motif after the transmembrane domain: a [RK][RK]X[RK][RK] motif, akin to that of *S. salmonicida*, though often followed with more N-terminal amino acids. When we relaxed this motif to be one amino acid pair shorter ([RK][RK]X[RK] or [RK]X[RK][RK]), we recover 99 and ± 10 of these “degraded” VSPs in *Hexamita* sp. SM and various *Trepomonas* species, respectively. In general, identification of VSPs in transcriptomes is challenging due to their variable patterns of expression. To address this concern, we ran the same analysis on the transcriptomes from *Giardia intestinalis* and *Spironucleus salmonicida* and recovered 92 VSP-like (and 67 CRMPs) sequences from *G. intestinalis* and 52 (and 127 CRMPs) from *S. salmonicida*, indicating that counts of VSP sequences in transcriptomes are to some extent likely underestimates. Similar to VSPs, leucine-rich repeat BspA-like proteins are expressed on the surface of the cell of other parasitic protists, including *Trichomonas* [37]. We also searched for these proteins in all diplomonad datasets analyzed here. We identified candidate sequences for leucine-rich repeat proteins in all diplomonads (Additional file 2: Table S2), with higher copy numbers of BspA-like proteins being found in *Trepomonas* spp. PPS6 (Fig. 2A).

### Virulence factors

We selected 12 additional putative virulence factors (Fig. 2A, see all trees in Additional file 3: Fig. S1) known from *Giardia intestinalis* and assessed their presence/absence in the diplomonad datasets. NADP-specific glutamate dehydrogenase (NADP-GDH), Extracellular nuclease (Ex. nuclease), Cathepsin B, Ornithine transcarbamylase (OTC), Arginine deiminase (ADI), Enolase, and Fructose-bisphosphate aldolase (FBPA) were present in all diplomonads except for the highly incomplete datasets of *Spironucleus vortens* and *S. barkhanus*. Thioredoxin was also found in most datasets, the exceptions being *S. vortens*, and *S. barkhanus*. The Cystatin, pyridoxamine 5’-phosphate oxidase (PNPO), and Bactericidal Permeability Increasing-like protein (BPI) genes were specific to *Giardia* spp., while tenascins were found in all parasitic diplomonads (*Giardia* spp. and *Spironucleus salmonicida*), *Hexamita* sp. SM, and four *Trepomonas* isolates (PIG, VLADA7, MIS2C, SOOS4; Fig. 2A).

### Disc protein signature

The 208 seed sequences putatively targeted to *Giardia*’s ventral disc [38–41] were assigned into 160 orthogroups (Additional file 4: Table S3). Several of these proteins are universally present in diplomonads, such as ankyrin repeat proteins, dyneins, kinases, and tubulins. Giardins have a patchy distribution across diplomonads. Mitotic spindle checkpoint protein MAD2 was present only in the parasitic diplomonads and *Hexamita* sp. SM. Seventy-three proteins from 65 orthogroups were exclusively present in *Giardia*.

### Energy generation and pyruvate metabolism

We assessed the presence/absence of 6 enzymes involved in anaerobic ATP production, including FeFe-hydrogenase and its maturases (HydA, HydE, HydF, and HydG), pyruvate:ferredoxin oxidoreductase (PFO), and two versions of ADP-forming acetyl-CoA-synthetase (ACS_ADP1 and ACS_ADP2), which are involved in anaerobic fermentation of pyruvate to acetyl-CoA to acetate (Additional file 5: Fig. S2). Each of these six enzymes appears to be present in all Hexamitinae diplomonads, while *S. barkhanus*,* G. intestinalis*,and* G. muris* are missing the FeFe-hydrogenase maturases (Fig. 2A).

### Horizontally transferred genes

We chose 8 previously identified HGTs [30] that are hypothesized to enhance the capability of cells to degrade bacterial cell walls (NlpC/P60, murein hydrolase, lysozyme) and synthesize deoxyribonucleotides (Ribonucleotide reductase (RNR), Adenosine deaminase (ADA), Deoxyuridine triphosphatase (DUT), Uracil phosphoribosyltransferase (UPRT), Purine nucleoside phosphorylase (PNP)) (Fig. 2A, see all trees in Additional file 6: Fig. S3). As such, they can be inferred to be direct adaptations to life outside a host. All the HGTs responsible for digestion of bacterial cell walls were present in all *Trepomonas* isolates. Murein hydrolase is also present in *Trimitus* spp. and lysozyme in *Trimitus* sp. FISH, *Gyromonas ambulans*, and *Hexamita* sp. SM. ADA was present in all diplomonads, except *Spironucleus barkhanus*, *S. salmonicida*, and *Giardia* spp. RNR was only present in *S. vortens*, NDL GhostHex, *Hexamita* sp. SM, *Trepomonas* sp. PC1, and *Trepomonas* sp. PPS6 VLADA7. DUT was present in *S. salmonicida*, *Hexamita* spp., and in most *Trepomonas* isolates. UPRT was specific to parasitic diplomonads, whereas PNP was present across all diplomonad isolates, excluding *S. barkhanus*.

## Discussion

Commensal and free-living diplomonads have been vastly understudied compared to their pathogenic relatives, despite providing a fascinating framework to study the evolution of these distinct lifestyles. Previously, Xu et al. [30] sequenced the transcriptome of *Trepomonas* sp. PC1 to explore the origin of a free-living lifestyle in diplomonads. They concluded that *Trepomonas* likely evolved from parasitic ancestors and re-acquired lost metabolic functions through horizontal gene transfers. We re-examined the hypothesis that the free-living lifestyle is secondarily acquired in diplomonads by inferring a robust phylogenomic framework from the transcriptomes of 14 newly sequenced diplomonad isolates from four genera and one novel lineage. We mapped the life histories of these newly sequenced diplomonad taxa onto this phylogenomic framework and have identified potential signals of parasitic ancestry in the transcriptomes of free-living taxa.

### Endobiotic and free-living status of studied diplomonad species and isolates

The endobiotic or free-living status of most diplomonads included in our study is reliable. *Giardia* spp. and *Spironucleus* spp. are endobionts, with most being well-studied and characterized as pathogens. *Gyromonas ambulans*, the novel diplomonad lineage GhostHex, and all *Trepomonas* spp. (with one possible exception) have been isolated from either freshwater or marine sediments and are free-living. Because the *Trepomonas agilis* PIG and MIS2C cultures were initiated from a manure pile on a pig farm and a waste water cleaning factory (respectively), it is conceivable that they could be endobionts. However, this seems unlikely, as to our knowledge *Trepomonas* has never been observed or isolated from any mammalian host. Thus, we are confident that *T. agilis* is free-living and that these isolates originated from contaminations from the environment.

**Fig. 3:**
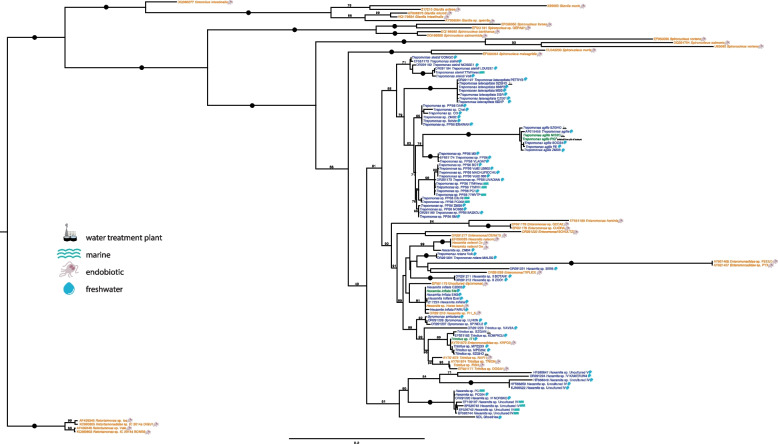
Maximum likelihood tree of Diplomonads based on the SSU rRNA gene. The tree was constructed in RAxML using GTRGAMMA as a model and 1000 non-parametric bootstrap replicates (values above the branches, dots denote 100% bootstrap support, all bootstrap values below 50% were removed). The color of the taxa corresponds to the lifestyle (as in the other figures): blue for free-living; orange for host-associated; green for potentially amphizoic. The origin of the isolates is denoted by the icons on the right side of the taxa

The normal living environment of *Trimitus* spp. and *Hexamita inflata*-like isolates is uncertain because they have been observed and cultured from both gut contents and from sediments. *Trimitus* sp. FISH was isolated directly from the gut of an unidentified catfish and is clearly endobiotic, as are all but one of the published *Trimitus* sp. isolates [6]. *Trimitus* sp. IT1 [6] and numerous other newly isolated *Trimitus* spp. that possess almost identical SSU rRNA gene sequences have been cultivated from anoxic sediments and are presumed to be free-living (Fig. 3). *Hexamita inflata* and numerous other *Hexamita inflata*-like cultures were all initiated from anoxic freshwater sediments and trophozoites can be directly observed in many of these sediments. This taxon is widely considered free-living [42]. However, all *H. inflata*-like isolates have an almost identical SSU rRNA gene sequence to that of *Hexamita* sp. observed within and cultured directly from the gut of a horse leech, *Haemopsis sanguisuga* (described as *Hexamita gigas*, [43]). In fact, the *Hexamita* sp. PARU isolate was cultured from sediments collected from the same small pond where the infected horse leech was living. Morphology and SSU rRNA gene sequences are consistent with all of these *Hexamita inflata*-like isolates belonging to the same taxon. These findings indicate that *Trimitus* spp. and *Hexamita inflata* may be capable of existing as facultative endobionts. We are further exploring this possibility (see below for extended discussion).

### A well-resolved phylogeny of diplomonads

Several previous studies have placed free-living diplomonads deep within their parasitic relatives; yet, all of these studies are based on either a single locus, or at best a few genes, and show low resolution and a severe lack of support for the relationships within diplomonads [5, 6, 8]. Only three previous studies have included the published transcriptome of *Trepomonas* sp. PC1 in their phylogenomic analyses; however, those studies were all focused on other parts of the metamonad phylogenetic tree (*Anaeramoeba* and *Carpediemonas*-like organisms [35, 36, 44]). Here, we have vastly increased the amount of available genome-scale data from free-living and commensal diplomonads, and our phylogenomic analyses (Fig. 1A) provide a solid evolutionary framework supporting these previous studies. The closest relatives to diplomonads are endobiotic vertebrate-inhabiting retortamonads. Likewise, the earliest diverging diplomonads are *Giardia* and *Spironucleus* spp., which are all endobionts. These data support previous assertions of a host-associated ancestral state for the entirety of Diplomonadida. Consistent with a reversion of habitat and life histories, all free-living taxa, including the monophyletic *Trepomonas*, are derived members of the crown group of diplomonads. A few endobionts and taxa with ambiguous native habitats are scattered among these free-living taxa in our phylogenetic and phylogenomic trees, rendering it difficult to ascertain the number of reversions to a secondarily free-living lifestyle that have occurred in the evolving history of diplomonads. Notably absent from our phylogenomic analyses are several known endobiotic diplomonad lineages, including *Enteromonas* spp. and several *Spironucleus* spp. Inclusion of these taxa in 4-gene and SSU phylogenetic analyses (Fig. 3) produces a topology, whose well-supported nodes are consistent with the phylogenomic tree. However, the unsupported nodes show that future phylogenomic analyses that include these missing taxa might produce topologies suggesting more switches to free-living or even secondarily derived endobiotic lifestyles (Fig. 1A, B).

### Hexamita diversity

Within the Hexamitinae, several morphological criteria differentiate members of the genera *Hexamita* from *Trepomonas*. While the members of each genera possess two nuclei with an associated quadri-flagellated kinetid, the arrangement of the flagella are dramatically different. Each flagellar apparatus of *Trepomonas* possesses an anterior flagellum and three trailing flagella that lie in a lateral groove along each side of the cell body [45]. *Hexamita* does not possess lateral grooves and one posterior flagellum from each kinetid traverses through the length of the cell body in a tube to emerge from the posterior end. Mazancová et al. [8] have shown that organisms commonly identified by light microscopy as *Hexamita* likely do not form a monophyletic group.

In phylogenomic analyses, our isolate NDL GhostHex branches sister to the *Hexamita*-*Gyromonas*-*Trimitus* clade, while in the four-gene phylogeny it branches sister to the *Trepomonas*-*Hexamita*-*Gyromonas*-*Trimitus* clade (Fig. 1A, B) and it likely represents a novel diplomonad lineage. The SSU rRNA gene phylogeny, containing the known breadth of diplomonad diversity, shows that NDL GhostHex is a close relative of, or even belongs to, *Hexamita* clade I as defined by Mazancová et al. [8]. *Hexamita* clade I branches away from *Hexamita inflata*, further confirming that NDL GhostHex belongs to a previously unidentified phylogenetic lineage of diplomonads. Indeed, we temporarily had in culture two isolates with a *Hexamita* phenotype that branch within this environmental clade (*Hexamita* sp. PC and PC004; Fig. 3). Although the backbone resolution of the SSU rRNA gene tree is poor, organisms with a *Hexamita* gross morphology branch in several distinctively different positions [8] (e.g., clades with NDL GhostHex, *Hexamita* sp. SM, and *Hexamita nelsoni*, Fig. 3). A formal description of NDL GhostHex, including morphology and ultrastructure studied by light and transmission electron microscopy, along with the inclusion of representatives of other *Hexamita* clades [8] into phylogenomic analyses will likely result in splitting the genus *Hexamita* into several separate genera.

### Evidence of parasitic ancestry of diplomonads

Genomes of parasites contain virulence factors, which are genes that are known or expected to facilitate diverse functions in host-parasite interactions; for example, avoiding the host immune system, attaching to host tissues, or modifying the host environment. If our phylogenetic analyses are correct and the ancestral diplomonad was an endobiont, it is reasonable to expect that some free-living taxa will still retain a fingerprint of the parasitic lifestyle within their genomes. Therefore, we selected 12 known virulence factors from *Giardia intestinalis* and attempted to identify these genes in all diplomonad datasets. In addition, *Giardia* spp. possess an adhesive disc that is used to attach to host epithelium. While this disc structure has not been observed in other diplomonads, we have attempted to identify homologs of the ± 200 proteins known to be localized to the disc in *Giardia*.

Gene presence is easy to interpret; however, it can be problematic trying to infer gene loss from transcriptomic datasets, as the condition of the organisms during RNA isolation might lead to specific gene expression profiles lacking certain genes. Yet, the studied organisms do not have complex life cycles or variable life stages that would lead to massive shifts in gene expression [35, 46]. Nevertheless, we conservatively define a gene as not present when it is missing from the majority of representatives of a particular lineage.

### VSPs and CRMPs

Cysteine-rich proteins are very common in diplomonad genomes and there are several categories defined based on their properties: Variant-specific surface proteins (VSPs), Cysteine-rich membrane proteins (CRMPs), Cysteine-rich secreted proteins (CRSPs), etc. Variant-specific surface proteins (VSPs), which are massively duplicated in *Giardia* and *Spironucleus* s*almonicida*, have been experimentally shown to aid in avoiding host immune responses [4, 47, 48]. Their typical structure is a long extracellular cysteine-rich domain, followed by a transmembrane domain and a short intracellular N-terminus with a highly conserved motif. This motif differs from lineage to lineage: CRGKA in *Giardia intestinalis*, CGRKG in *Giardia muris*, and [RK][RK]X[RK][RK] in *Spironucleus salmonicida*. The CRMPs are similar in structure to VSPs, but without the highly conserved motif on the N-terminus [49].

To explore our data for concrete evidence of parasitic ancestry in free-living lineages, we looked for the retention of VSPs across diplomonads and their relatives—*Carpediemonas*-like organisms, *Retortamonas* spp., *Chilomastix* spp., and *Barthelona* sp. (Fig. 2, Table 1, Additional file 1: Table S1). In our newly sequenced diplomonads, we did not identify any representatives of Dicer or Argonaute proteins, which in *Giardia*, are assumed to regulate VSP expression. This suggests a different manner of gene regulation, as seems to be the case in *S. salmonicida*. While all diplomonads possess a sizable number of cysteine-rich proteins, their relatives possess close to none, with the exception of their closest relatives. Specifically, the two *Retortamonas* strains and *Dysnectes brevis* possess a small number of CRMPs and one putative VSP protein each. Interestingly, typical VSP proteins appear to be specific to parasitic diplomonads, *Giardia intestinalis*, *G. muris*, and *Spironucleus salmonicida*, which were previously hypothesized to have independently evolved their VSPs, or at least the conserved motifs [18]. The apparent absence of VSPs in the other two *Spironucleus* species is likely due to a paucity of data, rather than an actual lack of these proteins (*S. vortens* and *S. barkhanus* are both represented by highly incomplete datasets). Although we were unable to identify a motif specific to any of the free-living diplomonads, we did find 28 putative VSPs in the transcriptome of *Hexamita* sp. SM with the [RK][RK]X[RK][RK] motif identical to that of *S. salmonicida.* Additionally, when we relaxed the motif criteria to include only three [RK] amino acid pairs, the number of putative VSPs in *Hexamit*a sp. SM grew to 99. Comparatively, only between 1 and 3 (3–34 with relaxed motif) putative VSP sequences with this motif were found in the free-living NDL GhostHex, *Gyromonas*, *Trimitus*, and *Trepomonas* species (Table 1). Based on currently available data, it seems that the [RK][RK]X[RK][RK] motif might be ancestral to Hexamitinae diplomonads.

Interestingly, when the numbers of putative VSPs are plotted along the diplomonad phylogeny, the pattern of differential VSP retention and loss appears to reflect the evolution of life strategies (Fig. 2A). The pathogenic *Giardia* spp. and *S. salmonicida* possess a large number of VSPs. *Hexamita* sp. SM, which was isolated from a pond as free-living, but has a nearly identical endobiotic congener ( [43], Fig. 3), retains at least 28 putative VSP sequences. In contrast, members of the free-living *Trepomonas* clade, *Gyromonas ambulans*, NDL GhostHex, and *Trimitus* appear to have lost the majority of their VSPs; the small number of remaining putative VSPs is intriguing evidence of their past presence. Notably, we did find a large number of cysteine-rich membrane proteins (CRMPs) in all diplomonad lineages. The distribution of CRMPs across the tree shows that the massive duplication and presence of cysteine-rich proteins is exclusive to diplomonads. Cysteine-rich proteins may have existed in their ancestors, but in much smaller numbers as compared to diplomonads. Taken together, CRMPs exist in multiple copies in all diplomonads, while the VSPs appear to be more specific to endobiotic and pathogenic species. This pattern suggests that CRMPs likely play a biologically important role across the diplomonads, perhaps in sensing or protection from the outer environment. The lack of expansion or loss of VSP genes in the endobiotic *Trimitus* spp. suggests, there is a different nature of its host interactions compared to that of *Giardia* and *Spironucleus*. Given the conservation of a specific motif in *S. salmonicida* and *Hexamita* sp. SM, it seems that VSPs were present in the genome of the Hexamitinae ancestor, but are not being maintained in free-living species, supporting the hypothesis that these lineages are secondarily free-living. Alternatively, the motif existed in the Hexamitinae ancestor functioning in any lifestyle (i.e., more akin to a CRMP protein), which would then have been independently derived in *Hexamita* sp. SM and *S. salmonicida*.

### Leucine-rich repeat proteins

Hirt et al. [37] identified leucine-rich repeat proteins (BspA-like proteins) on the surface of the parasite *Trichomonas vaginalis* and suggested that they might be utilized to mediate oxidative stress. We searched our data for all eleven different leucine-rich repeat domains from the Pfam database and identified six of them in several of our datasets (Additional file 2: Table S2). This would be expected, given that these proteins are involved in many cellular functions (for example in *Giardia*, the cyst wall proteins contain leucine-rich repeat domains). Interestingly, the BspA-like proteins (i.e., containing the Pfam domain LRR_5) are present in high copy number in *Trepomonas* spp., and several have a recognizable transmembrane domain, which suggests their localization on the cell surface (Fig. 2A, Additional file 2: Table S2). Free-living *Trepomonas* spp. are likely exposed to oxygen more frequently than endobiotic diplomonads, as an absence of oxygen in their free-living environments is less stable than in a host gut habitat. Therefore, these proteins could play a role mediating oxidative stress, like in *T. vaginalis*.

### Universal and specific virulence factors

We observed two distinct patterns in the retention of analyzed virulence factors across the diplomonads. First, some virulence factors are almost universally present across diplomonads, representing genes that are assumed to act as virulence factors in *Giardia*, but also possess some other basic metabolic function in free-living organisms. Indeed, these include relatively common housekeeping enzymes—for example, enolase, enzymes involved in the arginine deiminase pathway, and fructose-bisphosphate aldolase—and their presence in almost any eukaryotic cell would be unsurprising. Likewise, thioredoxin and NADP-glutamate dehydrogenase are enzymes involved in protection against reactive oxygen species. Since all diplomonads are anaerobes, these enzymes are expected to exist in free-living species as well. It is likely that most of these enzymes are moonlighting as virulence factors due to the experimental settings—for example, the differences between tissue cultures and a real gut environment—rather than being genuinely involved in pathogenesis.

Secondly, there are a few virulence factors that are present only in *Giardia* (Cystatin and BPI), or in *Giardia* spp., *Spironucleus*, and *Hexamita* sp. SM (tenascins). Cystatins are likely involved in host immune system modulation [50, 51] and BPI may serve as a regulator of bacterial growth [15, 52]. Cystatin and BPI seem to be specific to *Giardia* spp., while tenascins are also found in *Spironucleus* spp*.*, *Hexamita* sp. SM and a few *Trepomonas* isolates (see Additional file 7: Fig. S4). Animal tenascins are vertebrate-specific and are involved in cell migration and adhesion [53]. Diplomonad tenascin-like proteins share the EGF (Epidermal Growth Factor) binding domain with vertebrate tenascins, yet they appear to have evolved independently from cysteine-rich proteins [53]. They are hypothesized, without strong cell biological evidence, to bind to EGF receptors of host epithelial cells to help maintain the separation of individual epithelial cells [25, 54]. If for argument’s sake, we consider this to be a specific function of diplomonad tenascins, then they would be differentiated from other putative virulence factors, as they would be directly involved in pathogenicity.

This is consistent with their retention in pathogens, but not in free-living diplomonads. The retention of several copies of tenascins in *Hexamita* SM might be suggestive of the nature of the organism’s relationship with its potential host. To better understand the distribution of these virulence factors and their impact on the interpretation of lifestyle evolution, it is important to obtain data from other endobiotic/parasitic species. *H. nelsoni* is especially key to this, as it is a known pathogen that, based on SSU data, branches among the free-living taxa.

As with other virulence factors, many of *Giardia*’s adhesive disc proteins are well studied and possess additional cellular functions [38, 40, 41]—like tubulins, kinases, and ankyrin repeat proteins; thus, their presence cannot be considered specifically associated with the disc structure. There are 52 such proteins present in all major diplomonad lineages, while 73 proteins localized to the disc appear to be unique to *Giardia*. Interestingly, there are several disc-localized proteins that are variably present in other diplomonads. Giardin proteins are an illustrative example: alpha-3 and alpha-6 giardin homologs were found in several Hexamitinae diplomonads, while alpha-11 giardin is present only in *Hexamita* sp. SM and alpha-17 is only in *Giardia*. From the distribution pattern, it appears that the *Giardia* disc is composed of both unique and specific proteins to *Giardia* and repurposed proteins of older evolutionary origins. Knowing the precise molecular/cellular function of some of these proteins in Hexamitinae diplomonads will likely provide further insight into the evolution of the *Giardia* disc.

### HGT candidates and a secondarily free-living lifestyle

In the newly sequenced transcriptomes, we investigated the HGTs that were hypothesized to enable the adaptation to a secondarily free-living lifestyle [30, 55] for their presence/absence, copy number, and likely ancestry. The first group of these genes, those that are likely involved in the digestion of bacterial cell walls, such as lysozyme, NlpC/P60, and murein hydrolase (Fig. 2A, Additional file 6: Fig. S3), appear to be present only in free-living (mostly in *Trepomonas* spp.) isolates and are present in a large number of copies per isolate, suggesting frequent gene duplication post HGT gain. Phylogenetic analyses of lysozyme and NlpC/P60 genes demonstrate their presence in other metamonads that branch separately from *Trepomonas* spp.; however, the resolution of the tree is too low to make any strong conclusions about common or independent HGTs (see Additional file 6: Fig. S3). These genes were not present in parasitic *Giardia* spp. or *Spironucleus* spp. This, coupled with their high copy number within free-living isolates likely indicates their importance to free-living diplomonads.

Genes encoding proteins involved in the metabolism of nucleotides—such as Ribonucleotide reductase (RNR), which is responsible for the reduction of ribonucleotides to deoxyribonucleotides [56]; Adenosine deaminase (ADA) that can shuttle inosine into purine metabolism making organisms less dependent on scavenging from the host [30]; Deoxyuridine triphosphatase (DUT) that synthesizes the conversion of dUMP into dTMP, the direct precursor of thymidine nucleotides [55]; Uracil phosphoribosyltransferase (UPRT), which creates UMP from uracil, making organisms more flexible in pyrimidine salvage [55]; and Purine nucleoside phosphorylase (PNP), an enzyme of the nucleotide salvage pathway that metabolizes inosine and guanosine to hypoxanthine and guanine, respectively [55] (see Additional file 6: Fig. S3)—tend to be less abundant and their presence/absence among free-living and endobiotic species is spotty. It was previously believed that the eukaryotic class of RNR was lost in the common ancestor of Diplomonadida, and then Hexamitinae regained anaerobic RNR, with subsequent loss in *S. salmonicida* [55]; however, after extending the taxon sampling, our data show that RNR was likely gained independently by the *Trepomonas*-*Trimitus*-*Gyromonas*-*Hexamita* clade, *Spironucleus vortens*, and other representatives of free-living diplomonads. ADA, which has previously been considered an independent gain by *Trepomonas*, appears to be an ancestral HGT to all Metamonada, followed by differential loss in *Giardia* and *Spironucleus salmonicida.* DUT is specifically present in Hexamitinae diplomonads, suggesting its origin by a single HGT event, as suggested previously by Jimenez-Gonzales and Andersson [55]. PNP, which is a key enzyme of the purine salvage pathway, is present across all eukaryotes including diplomonads, whereas UPRT, essential for pyrimidine salvage, is an ancestral HGT to Metamonads and has been lost in the free-living diplomonads.

### Anaerobic energy generation

Diplomonads, like many other anaerobic eukaryotes, have adapted to their anoxic lifestyle by anaerobically generating ATP through metabolizing pyruvate to acetyl-CoA to acetate by substrate-level phosphorylation (among other possible pathways). Mitochondrial organelles have been largely reduced in diplomonads. *Giardia intestinalis* possesses mitosomes that are only known to function in Fe-S cluster synthesis and associated reactions [13, 57]. *Giardia* also generates ATP through the cytosolic arginine deaminase pathway, which in turn may serve to reduce host innate immunity by depleting arginine [58]. We have identified key enzymes of both anaerobic pyruvate metabolism and the arginine deaminase pathway in most diplomonads. *Spironucleus salmonicida* seems to possess a more complex mitochondrial organelle than *Giardia*, as it has been shown to be involved in ATP generation and to generate molecular hydrogen, characterizing it as a hydrogenosome [11]. Organellar ATP generation in *S. salmonicida* is possible due to the presence of a unique acetyl-CoA synthetase enzyme that is localized to the hydrogenosome. We found orthologs of this hydrogenosomal acetyl-CoA-synthetase in *Trimitus*, *Hexamita* sp. SM, and a few *Trepomonas* isolates (Fig. 2A), indicating its origin in the ancestor of Hexamitinae diplomonads and suggesting that some of them might contain hydrogenosome-like organelles (Additional file 5: Fig. S2).

### Evolution of parasitic and free-living lifestyles within diplomonads

If we assume only one direction of lifestyle change within the clade of diplomonads, then the phylogenomic tree presents two possible scenarios for the evolution of parasitic and free-living lifestyles: (1) an endobiotic/parasitic ancestor of diplomonads, followed by three independent transitions to a free-living lifestyle; or (2) a free-living ancestor of diplomonads, followed by up to four independent transitions to an endobiotic lifestyle. A reversal from parasitism to a free-living lifestyle is believed to be an extraordinarily rare, if not impossible event, as the parasitic lifestyle is often considered a terminal evolutionary path [33, 59]. Host reliance commonly leads to genomic modifications that include the loss of essential metabolic pathways, which is thought to render parasites (and other symbionts) incapable of returning to a free-living lifestyle. However, diplomonads represent one of the few lineages where the transition from parasitic back to a free-living lifestyle has been suggested [30].

In this context, two previous key studies explored the evolution of a free-living diplomonad. Xu et al. [30] sequenced and analyzed the transcriptome of *Trepomonas* sp. PC1, and identified several HGTs unique to *Trepomonas* sp. PC1 that appear to have helped it overcome the loss of essential functions required for a free-living lifestyle. More recently, Jimenez-Gonzales and Andersson [55] analyzed the gene content of diplomonad genomes and the transcriptome of *Trepomonas* sp. PC1, and identified several genomic signatures characteristic of parasitism that were present in the last common ancestor of diplomonads, including streamlined genomes and metabolic capabilities. Both studies support the parasitic ancestry of diplomonads. However, our extended taxonomic sampling of diplomonads complicates this story, as it suggests several independent switches from, or to, a parasitic lifestyle. Like the previous studies, our results could support a parasitic ancestry followed by reversals to a free-living lifestyle. Both VSPs and tenascins were likely present in the diplomonad common ancestor, as they are still retained in pathogens and were independently lost/reduced in the free-living taxa (Fig. 2A). *Hexamita* sp. SM also possesses both VSPs and tenascins and its SSU rRNA gene sequence is nearly identical to that of an endobiont of horse leeches, supporting its likely endobiotic nature. The branching of the VSP and tenascin-lacking endobiotic *Trimitus* sp. among the free-living taxa further complicates this scenario. Therefore, our results highlight the need for a more nuanced interpretation of transitions between parasitism and free-living lifestyles in diplomonads (Fig. 2B).

When discussing a reversal from parasitism to free-living lifestyle in diplomonads, we must not assume only two distinct states but take into account that the interactions of endobionts with, and the extent of dependence upon their hosts, likely form a continuum [60]. This can range from highly specialized pathogens with complex host interactions, through harmless commensals that simply occupy a unique niche, feeding on the host’s prokaryotic microbiome and having marginal direct interactions with the host, to mutualists that can help modulate the host microbiome or immunity [61]. Moreover, free-living diplomonads reside in anaerobic sediments, which are generally rich in prokaryotes and can experience swift environmental changes. In many respects, such habitats represent a similar ecological niche to the colon of an animal, especially from the perspective of a diplomonad [33]. Together with previous studies, our phylogenomic and comparative analyses, as well as the variability in lifestyles, hosts, and habitats observed across the diplomonads suggest that their ancestor was an endobiont—possibly parasitic, but likely not a highly specialized parasite (Fig. 2B). *Giardia* and *Spironucleus* then evolved separately to become highly specialized pathogens, while the ancestors of *Trepomonas*, *Gyromonas*, and NDL GhostHex transitioned towards a free-living lifestyle (Fig. 2B). *Hexamita* spp. and *Trimitus* spp. either remained endobiotic (and our isolates are artifacts of sampling contaminations), or they might represent amphizoic lineages capable of both free-living and endobiotic lifestyles. While the latter might seem unlikely, it is further supported by the pattern of specific environments from which these diplomonads have been isolated. *Giardia* and *Spironucleus* are not commonly detected in sediments, while the free-living *Trepomonas* spp. are not generally isolated from host environments (with the exception of two reported isolates from insects [8]). On the other hand, representatives from the genera *Hexamita* and *Trimitus* have been isolated from both free-living (anaerobic sediments) and host associated environments (Fig. 3). This would suggest that either *Hexamita* or *Trimitus* are facultative endobionts/free-living (i.e., can exist as endobionts and as free-living organisms), or that these genera contain species that are endobiotic and others that are free-living. Interestingly, *Gyromonas ambulans*, which has only been described as free-living, is closely related to *Trimitus*, suggesting a relatively recent switch in lifestyle preferences.

To better understand the evolution of transitions between parasitic and free-living lifestyles, it will be necessary to obtain complete genome sequences from additional free-living diplomonads to confirm our results regarding gene presence/absence. Since HGTs have been suggested to facilitate expansion of the metabolic potential of secondarily free-living diplomonads, large-scale analyses of horizontally transferred genes will also help resolve this complex evolutionary scenario. Lastly, culturing experiments of potentially amphizoic species in free-living and endobiotic settings along with differential expression analyses would clarify the ability of these species to survive both as free-living and as endobionts.

## Conclusions

Using transcriptome sequencing, we have more than tripled the taxon sampling of diplomonads available for genome-scale analyses. Phylogenomic analyses of these data have shown that transitions between endobiotic and free-living lifestyles have likely occurred multiple times during the evolutionary history of diplomonads, as neither free-living nor endobiotic diplomonads form a monophyletic group. Instead, the free-living taxa form several clades within parasitic diplomonads. Moreover, traces of a genetic toolkit for host-associated lifestyle in free-living diplomonads corroborate our inference of their endobiotic ancestry. Based on these results, we have concluded that the extant diversity of diplomonads have evolved from a non-specialized endobiont, with some taxa becoming highly specialized parasites, others becoming free-living, and some becoming capable of both free-living and endobiotic lifestyles.

## Methods

### Isolation, culturing, RNA isolation, and sequencing

Fourteen diplomonads (see Additional file 1: Table S1, [8]) were isolated and maintained in ATCC 802 Sonneborn’s *Paramecium* medium (*Gyromonas ambulans*, *Trepomonas* spp.), 1:1 mixture of 3%LB:802 with heat-inactivated horse serum (5% final concentration; new diplomonad lineage GhostHex), or TYSGM-9 medium (*Trimitus* sp. FISH, *Hexamita* spp.). Cultures were maintained in the dark under microaerophilic conditions at 18 °C. *Trepomonas* sp. PIG was isolated from a manure pile (Sanpong village, Phan district, Chiang Rai Province, Thailand), and *Trimitus* sp. FISH was isolated from the gut of a Siluriformes catfish (from a river in Chiang Rai Province, Thailand). All other isolates used for phylogenomic analyses were isolated from freshwater anoxic sediments.

Cultures were independently scaled up to 500 mL volume for RNA isolation. Upon satisfactory cell density, cultures were centrifuged at 900* g* for 7 min at room temperature (~ 22 °C). The supernatant was removed, each cell pellet was resuspended in 2 mL of fresh media, transferred into a 2-mL Eppendorf tube and pelleted once more at 1100* g* for 7 min at room temperature (~ 22 °C). Total RNA was isolated from cell pellets with TRI Reagent (SigmaAldrich) following the manufacturer’s protocol, using either BCP (1–bromo–3–chloropropane) or chloroform to separate the phases. The resulting RNA was quantitated by NanoDrop (Thermo Scientific) and Qubit (Invitrogen) and shipped to Macrogen Inc. (South Korea) for TruSeq stranded mRNA library preparation and sequencing. Each library was sequenced on an Illumina NovaSeq, producing 30 million 150-bp paired-end reads.

### Data assembly and cleaning

Sequencing reads were checked for quality using FastQC v. 0.11.9 [62]. Low quality bases (Q < 30) and adapters were removed using Trimmomatic v. 0.38 [63]. The remaining reads were assembled using rnaSPAdes v. 3.13 [64]. Specifying the non-canonical Hexamitinae genetic code, Transdecoder v. 5.5.0 (https://github.com/TransDecoder/TransDecoder) was used for ORF prediction and translation to amino acids. To remove obvious contamination from other organisms, each Hexamitinae transcript was searched using diamond BLASTP against a non-redundant NCBI database (with e-value set to 1e − 10 and more-sensitive search parameter). Sequences that had no in-frame stop codons and hit bacteria or non-metamonada eukaryotes with > 50% identity over 100 amino acids (or 50% length of the transcript) were removed as potential contaminations. BUSCO (Benchmarking Universal Single-Copy Orthology) scores against eukaryotic lineages [65] was used to estimate individual dataset completeness against the eukaryotic lineages (Fig. 2A, Additional file 8: Fig. S5). Both full and cleaned assemblies are available in the Figshare repository [66].

### Phylogenomics/phylogenetics

All publicly available Metamonada genomes/transcriptomes were considered for initial ortholog selection. The Phylofisher [67] script fisher.py was used to identify potential orthologs from all metamonads; informant.py provided information about the number of potential orthologs identified for each taxon by gene; and working_dataset_constructor.py was run to compile single gene fasta files for all potential orthologs and paralogs for these data and all others in the publicly provided Phylofisher database, which is comprised of taxa spanning the eukaryotic tree of life. Each individual gene was filtered for sequencing errors and non-homologous sites using PREQUAL v. 1.02 [68], aligned using MAFFT v. 7.453 [69] (-globalpair –maxiterate 1000), and alignment uncertainty and errors were filtered using DIVVIER v. 1.01 (https://github.com/simonwhelan/Divvier) (–partial -mincol 4 -divvygap). Trees were constructed from the trimmed alignments using IQ-TREE v. 1.6.12 [70] under the LG + C20 + F + G model, and 100 rapid bootstrap replicates for each gene were estimated using RAxML v. 8.2.12 with the PROTGAMMALG4X model [71]. Bootstrap support was mapped to each tree and the resulting trees were then manually inspected to remove paralogous sequences and contamination.

Once all single gene trees were manually parsed, the chosen orthologs were incorporated into the Phylofisher database [67] and an ortholog-only dataset was constructed from the 188 genes, which included metamonad taxa. The Phylofisher script matrix_constructor.py was used under default settings to align and concatenate the orthologs, producing the final phylogenomic matrix comprised of 188 genes (46,422 sites) and 45 taxa. This dataset was subjected to maximum likelihood analysis in IQ-TREE (LG + C60 + F + G) with 1000 PMSF bootstrap replicates. Bayesian inference was conducted in PhylobayesMPI v. 1.5 [72] under the CAT-GTR model with 4 chains for each dataset run for more than 12,000 generations each with chains nearing convergence upon the same topology (burnin = 10%; maxdiff = 0.162147). The final Phylofisher database that includes all considered sequences (i.e. orthologs and paralogs) is available in the Figshare repository [66].

To assess congruence with the phylogenomic results, we created and analyzed two datasets with dramatically increased taxon sampling: a small subunit rRNA (SSU rRNA)-only dataset and a 4-gene dataset based on concatenated HSP90, EF1-α, β-tubulin, and SSU rRNA genes. For the individual protein datasets in the 4-gene dataset, we used the same procedure for assembling and analyzing the phylogenomic dataset, while the SSU dataset was assembled manually and aligned using MAFFT v. 7.453 [69] (with -globalpair –maxiterate 1000 settings) followed by manual masking of unreliably aligned regions. The protein-gene amino acids and SSU nucleotides were then analyzed in separate partitions using C60 + LG + F + G and GTR + G models, respectively (Bayesian analysis was not performed). 1000 bootstrap replicates were computed using LG4X + G + F model for the protein partition and GTR + G model for the 18S partitions. For the SSU rRNA-only analysis, the SSU rRNA gene from each transcriptome assembly was retrieved, while 43 other isolates were PCR amplified from monoeukaryotic cultures (see Additional file 1: Table S1). These were added to all the diplomonads plus a subset of the vertebrate retortamonad SSU rRNA sequences in the EukRef dataset [73]. Sequences were then aligned using MAFFT v. 7.453 [69] (with -globalpair –maxiterate 1000 settings), poorly aligned regions were manually removed, and the ML tree was inferred using RAxML v. 8.2.12 with GTRGAMMA as a model and 1000 non-parametric bootstrap replicates [71].

### Removal of fast evolving and heterotacheous sites, random gene subsampling, and AU test

The Phylofisher scripts fast_site_remover.py, heterotachy.py, and random_resampler.py were used to subsample our 45-taxon 188-gene alignment (section Phylogenomics/Phylogenetics in Methods) to generate datasets for downstream analyses. The fastest evolving sites were sequentially removed in 3000 site chunks, generating new alternative datasets at each step until all sites were removed. Similarly, the most heterotacheous sites were removed in a stepwise fashion, 3000 sites at a time, producing iteratively smaller datasets until no further sites could be removed. Genes from our 45 taxon datasets were randomly subsampled in sets of 20, 40, 60, and 80% of the complete dataset, under the default 95% confidence interval setting as in Salomaki et al. [46]. Bootstrap support (100 replicates) for all datasets were generated using RAxML v. 8.2.12 under the PROTGAMMALG4X model. AU test was computed in IQ-TREE using the maximum likelihood topology, a constraint topology of monophyletic free-living diplomonad isolates plus 100 background topologies generated during ML search by IQ-TREE.

### Variant-specific surface protein identification

Identification of putative variant-specific surface proteins (VSPs) was conducted by first identifying all cysteine-rich (> 10% cysteine) proteins in each assembly. These were analyzed with TMHMM v. 2.0 [74], and proteins lacking transmembrane domains were removed. Next, proteins were filtered for those containing > 5 CXC or CXXC motifs, which have been described as a key feature of VSPs [24]. The remaining proteins were investigated for remnants of conserved motifs that have been identified adjacent to the transmembrane domain in *G. intestinalis* (CRGKA), *G. muris* (GCRGK), and *S*. *salmonicida* ([RK][RK]X[RK][RK]) VSPs. Proteins with less than 100 amino acids after the transmembrane domain were found and an identifier (“XXXXXXZZZZZZXXXXXX”) was inserted at the end of the transmembrane domain as a reference to align sequences on the domain. Sequences were then manually aligned on the inserted identifier and inspected for remnants of conserved motifs.

### Identification of other genes of interest

Seed sequences (from *G. intestinalis* and *Trepomonas* sp. PC1) of 12 genes for known virulence factors and 8 HGTs inferred to be linked to adaptation back to a free-living life strategy [30] were used as blast queries against a database consisting of all available Fornicata datasets, including the newly sequenced diplomonads; all sequence-hits with an e-value 1e − 10 or better were recovered as candidate sequences. Candidate Fornicata sequences were blasted against a non-redundant NCBI database and the top five sequences were retrieved from the BLASTP output and added to the Fornicata sequences, forming the preliminary dataset. This preliminary dataset was then filtered using PREQUAL v. 1.02 [68] (with default options) to remove non-homologous regions present in the low-quality sequences and aligned with MAFFT v. 7.453 [69] (with -globalpair –maxiterate 1000 settings). Aligned sequences were subjected to partial filtering using Divvier v. 1.01 with options divvygap and mincol set to 4 (https://github.com/simonwhelan/Divvier). Ambiguously aligned regions were excluded with BMGE v. 1.12 [75] (gap threshold 3%), and sequences that contained more than 90% gaps were excluded. The remaining sequences were then pulled from the untrimmed divvier output and retrimmed using trimAl v. 1.4 [76] (gap threshold 1%). Trees were computed with IQ-TREE v. 1.6.12 [70], using the LG + C20 + F + G model, and 100 rapid bootstrap replicates were generated using RAxML v. 8.2.12 with PROTGAMMALG4X model [71]. For 7 trees of genes involved in anaerobic energy generation, we used the preexisting phylogenetic datasets from Leger et al. [35] and Salomaki et al. [46] as starting datasets. Using seed sequences from these trees, candidate sequences were recovered by BLASTP (e-value 1e − 10) and added to a corresponding starting dataset. Initial trees were constructed as specified above. All trees were manually inspected to remove sequences that represented other genes, etc. After inspection and removal of xenolog sequences, the final trees were constructed as described above.

To assess the presence/absence patterns of disc proteins across diplomonad lineages—known as major virulence factors in *Giardia—*all diplomonad datasets were searched with HMM profiles. First, OrthoFinder v. 2.5.4 [77] was used to assign the predicted proteins of all diplomonad datasets to orthogroups. Known *Giardia* cytoskeleton and disc proteins were assigned to orthogroups along with other diplomonads. Next, the sequences of each orthogroup containing a target sequence were used to build HMM profiles with hmmer v. 3.3 [78] for the detection of homologs in diplomonad lineages. All matches with an e-value below 1e − 10 were used as queries for reciprocal BLASTP searches against the *Giardia* proteome to exclude putative paralogous and counterfeit matches. Recovered matches are shown in Additional file 4: Table S3.

Similarly, HMM profiles of eleven different Leucine-rich repeat domains were downloaded from the Pfam-A database and the software package hmmer v. 3.3 [78] was used to search for these domains in all diplomonad datasets used in this study. All hits with an e-value lower than 1e − 10 were retained and used as input into the TMHMM v. 2.0 [74] to identify transmembrane domains.

## Supplementary Information

Additional file 1: Supplemental Table S1. Overview of the isolates included in the analysis and accession numbers for the raw sequence, and SSU rRNA gene sequence data used in the analysis. Explanations: DE – Germany, AT – Austria, NO – Norway, ZM– Zambia, IT – Italy, TH – Thailand, CZ – Czech Republic, RI – Rhode Island, USA, NY – New York, USA, and MA – Massachusetts, USA.

Additional file 2: Supplemental Table S2. Copy number of the Leucin-rich repeat proteins in the transcriptomes used in this study.

Additional file 3: Fig. S1. Maximum Likelihood trees showing selected virulence factors. IQ-TREE with C20+LG+G+F model, statistical support was inferred from 100 rapid bootstrap replicates computed in RAxML with LG4X+G model. Host associated diplomonads are marked in orange, free-living in blue, and green shows diplomonads whose lifestyle is uncertain. Other eukaryotes are colored in red. To improve readability of the trees, large bacterial or eukaryotic clades were collapsed. Sequences that are possible bacterial contamination were marked in grey.

Additional file 4: Supplemental Table S3. Presence/absence of the cytoskeleton and disc proteins in the transcriptomes used in this study. The presence of the protein is indicated by the color blue.

Additional file 5: Fig. S2.Maximum Likelihood trees of the selected genes involved in energy generation and pyruvate metabolism. Trees were computed using IQ-TREE with C20+LG+G+F model, statistical support was inferred from 100 rapid bootstrap replicates computed in RAxML with LG4X+G model. Host associated diplomonads are marked in orange, free-living in blue, and green shows diplomonads whose lifestyle is uncertain. Other eukaryotes are colored in red.

Additional file 6: Fig. S3. Maximum Likelihood trees of the selected HGT datasets. Trees were computed using IQ-TREE with C20+LG+G+F model, statistical support was inferred from 100 rapid bootstrap replicates computed in RAxML with LG4X+G model. Host associated diplomonads are marked in orange, free-living in blue, and green shows diplomonads whose lifestyle is uncertain. Other eukaryotes are colored in red. To improve readability of the trees, large bacterial or eukaryotic clades were collapsed.

Additional file 7: Fig. S4. Maximum likelihood tree of the tenascin gene. Tree was computed using RAxML with LG4X+G model and statistical support was inferred from 100 non-parametric bootstrap replicates. Host associated diplomonads are marked in orange, free-living in blue, and green shows diplomonads whose lifestyle is uncertain. Other eukaryotes are colored in red.

Additional file 8: Fig. S5. Bar chart summarizing BUSCO results against eukaryotic lineages generated using generate_plot.py. An asterix in front of a species’s name denotes either incomplete (*S. barkhanus*, *S. vortens*) or contaminated (*Trepomonas *sp. PPS6 DAR, *Trepomonas *sp. PPS6 Vlada7) datasets.

## Data Availability

Datasets supporting the conclusions of this article are available in the Figshare repository under 10.6084/m9.figshare.26348908 [66]. Sequence data generated for this study are deposited in the NCBI SRA archive under accession PRJNA1066712 [79]. Phylogenomic single-gene alignments, including both orthologs-only and orthologs and paralogs, as well as other phylogenetic datasets are deposited in Figshare repository under 10.6084/m9.figshare.26348908 [66]. SSU rDNA sequences are deposited in GenBank under accessions: OR665824, OR670371-OR670422 (see Additional file 1: Table S1, section New SSU rRNA gene sequence data).

## Abbreviations

HGT: Horizontal gene transfer
SSU rRNA: Nuclear small subunit rRNA gene
VSPs: Variant-specific surface proteins
PNPO: Pyridoxamine 5’-phosphate oxidase
BCP: 1–Bromo–3–chloropropane
ML: Maximum likelihood
BI: Bayesian inference
NDL: Novel diplomonad lineage
AU: Approximately unbiased
TM: Transmembrane domain
CRMP: Cysteine-rich membrane proteins
CRSP: Cysteine-rich secreted proteins
NADP-GDH: NADP specific glutamate dehydrogenase
Ex. nuclease: Extracellular nuclease
OTC: Ornithine transcarbamylase
ADI: Arginine deiminase
FBPA: Fructose bisphosphate-aldolose
PNPO: Pyridoxamine 5’-phosphate oxidase
BPI: Bactericidal Permeability Increasing-like protein
RNR: Ribonucleotide reductase
ADA: Adenosine deaminase
DUT: Deoxyuridine triphosphatase
UPRT: Uracil phosphoribosyltransferase
PNP: Purine nucleoside phosphorylase
PMSF: Posterior mean site frequency
BP: Bootstrap support
PP: Posterior Probability
