# Supplementary material for: Expanded gene and taxon sampling of diplomonads shows multiple switches to parasitic and free-living lifestyle
Source: BMC Biol. 2024 Sep 27;22:217. doi: 10.1186/s12915-024-02013-w (PMC11437800; doi:10.1186/s12915-024-02013-w)
Supplement: Supplementary file 1 — Additional file 1: Supplemental Table S1. Overview of the isolates included in the analysis and accession numbers for the raw sequence, and SSU rRNA gene sequence data used in the analysis. Explanations: DE – Germany, AT – Austria, NO – Norway, ZM– Zambia, IT – Italy, TH – Thailand, CZ – Czech Republic, RI – Rhode Island, USA, NY – New York, USA, and MA – Massachusetts, USA. [file 12915_2024_2013_MOESM1_ESM.docx]

| **Isolate** | **Location with Lat long** | **Data Source** | **Sampling environment** | **Known Contaminants** |
| --- | --- | --- | --- | --- |
| Newly Generated Data | | | | |
| *Gyromonas ambulans* | (Mazancová et al. 2023) | PRNJ1066712 | Free-living:  freshwater |  |
| *Hexamita* sp. SM | Schwarzenbruck, DE  49.3553° N  11.2297° E | PRNJ1066712 | Free-living:  freshwater |  |
| Hexamitidae sp. GhostHex  (NDL GhostHex) | Cape Cod, MA, USA.  41.640327, -70.415700 | PRNJ1066712 | Free-living:  freshwater |  |
| *Trepomonas agilis* MIS2C | (Mazancová et al. 2023) | PRNJ1066712 | Sewage treatment facility: freshwater |  |
| *Trepomonas agilis* PIG | Sanpong village, TH | PRNJ1066712 | Free-living:  Manure pile |  |
| *Trepomonas agilis* SOOS4 | (Mazancová et al. 2023) | PRNJ1066712 | Free-living:  freshwater |  |
| *Trepomonas* sp. PPS6 BOT | Prague, CZ  50.04° N  14.24° E | PRNJ1066712 | Free-living:  freshwater |  |
| *Trepomonas* sp. PPS6 DAR | (Mazancová et al. 2023) | PRNJ1066712 | Free-living:  freshwater |  |
| *Trepomonas* sp. PPS6 ERAWAN | (Mazancová et al. 2023) | PRNJ1066712 | Free-living:  freshwater |  |
| *Trepomonas* sp. PPS6 MACHUPICCHU | (Mazancová et al. 2023) | PRNJ1066712 | Free-living:  freshwater |  |
| *Trepomonas* sp. PPS6 VLADA7 | (Mazancová et al. 2023) | PRNJ1066712 | Free-living:  freshwater | Ciliates |
| *Trepomonas steinii* CONGO | (Mazancová et al. 2023) | PRNJ1066712 | Free-living:  freshwater | *Naegleria* |
| *Trimitus* sp. IT1 | (Kolisko et al. 2008) | PRNJ1066712 | Free-living:  freshwater |  |
| *Trimitus* sp. FISH | Sanpong village, TH | PRNJ1066712 | Endobiotic: gut of Siluriformes catfish |  |
| Publicly Available Data | | | | |
| *Aduncisulcus paluster* |  | SRR3741808 |  |  |
| *Anaeramoeba flamelloides* |  | https://doi.org/10.6084/m9.figshare.12205517.v1 |  |  |
| *Anaeramoeba ignava* |  | https://doi.org/10.6084/m9.figshare.12205517.v1 |  |  |
| *Barthelona* sp. PAP20 |  | https://doi.org/10.5061/dryad.3tx95x6bn |  |  |
| *Carpediemonas membranifera* |  | SRR3734914 |  |  |
| *Chilomastix caulleryi* |  | SRX1890443 |  |  |
| *Chilomastix cuspidata* |  | SRR3734934 |  |  |
| *Dysnectes brevis* |  | SRR3742548 |  |  |
| *Ergobibamus cyprinoides* |  | SRX1890446 |  |  |
| *Giardia intestinalis* |  | GCF_000002435.1_GL2 |  |  |
| *Giardia intestinalis* RNA |  | SRR10662891 |  |  |
| *Giardia muris* |  | PRJNA524057 |  |  |
| *Kipferlia bialata* |  | GCA_003568945.1 |  |  |
| *Monocercomonoides exilis* |  | https://giardiadb.org |  |  |
| *Paratrimastix pyriformis* |  | GAFH01000000 |  |  |
| *Pentatrichomonas hominis* |  | SRR4896704 |  |  |
| *Retortamonas* cf. *caviae* |  | SRR12827909 |  |  |
| *Retortamonas dobelli* |  | SRR12827910 |  |  |
| *Spironucleus barkhanus* |  | NCBI GW585169-GW589878 |  |  |
| *Spironucleus salmonicida* |  | GCA_000497125.1 |  |  |
| *Spironucleus salmonicida* RNA |  | SRR948595 |  |  |
| *Spironucleus vortens* |  | JGI |  |  |
| *Tetratrichomonas gallinarum* |  | SRR2989159 |  |  |
| *Trepomonas* sp. PC1 |  | SRR2079337 |  |  |
| *Trichomitus batrachorum* |  | SRX2052874 |  |  |
| *Trichomonas gallinae* |  | SRX2052872 |  |  |
| *Trichomonas tenax* |  | SRX2052871 |  |  |
| *Trichomonas vaginalis* |  | http://trichdb.org/common/downloads/Current_Release/TvaginalisG3/fasta/data/ |  |  |
| *Trimastix marina* |  | SRR4017103 |  |  |
| *Tritrichomonas foetus* bovine |  | SRX540117 |  |  |
| *Tritrichomonas foetus* feline |  | SRX540971 |  |  |
| *Tritrichomonas foetus* genome |  | GCA_001839685.1 |  |  |
| *Tritrichomonas foetus* porcine |  | SRR1948019 |  |  |
| New SSU rRNA gene sequence data | | | | |
| *Hexamita* sp. PC004 | Prince Cove, MA, USA.  41.641629, -70.413456 | OR670385 | Free-living: marine |  |
| *Hexamita* sp. PC | Prince Cove, MA, USA.  41.641629, -70.413456 | OR670386 | Free-living: marine |  |
| *Hexamita inflata* SM | Schwarzenbach Moore, DE.  49.355350, 11.229707 | OR670381 | Free-living: freshwater |  |
| *Hexamita inflata* PARU1 | Ceske Budejovice, CZ.  48.975930, 14.447659 | OR670415 | Free-living: freshwater |  |
| *Hexamita* sp. HorseLeech | Ceske Budejovice, CZ.  48.975930, 14.447659 | [OR665824](https://www.ncbi.nlm.nih.gov/nuccore/OR665824) | Endobiotic: gut of Horse Leech, *Haemopis sanguisuga* |  |
| *Hexamita inflata* CZ002 | Cejkovice, CZ.  49.001134, 14.376361 | OR670417 | Free-living: freshwater |  |
| *Hexamita inflata* Ezel | Ezelsdorf, DE.  49.339677, 11.327147 | OR670416 | Free-living: freshwater |  |
| *Hexamita inflata* SM3 | Schwarzenbach Moore, DE.  49.355350, 11.229707 | OR670418 | Free-living: freshwater |  |
| *Hexamita nelsoni* Oe | Heligoland, DE.  54.182166, 7.879809 | OR670412 | Endobiotic: gut of European oyster, *Ostrea edulis* |  |
| *Hexamita nelsoni* Cv | Warham, MA, USA.  41.750554, -70.700798 | OR670413 | Endobiotic: gut of American oyster, *Crassostrea virginica* |  |
| *Hexamita* sp. ZMB4 | Close to Watopa, ZM, Africa.  -14.0266733, 23.6952603 | OR670414 | Free-living: freshwater |  |
| Hexamitidae sp. GhostHex  (NDL GhostHex) | Cape Cod, MA, USA.  41.640327, -70.415700 | OR670371 | Free-living: freshwater |  |
| *Trepomonas latecapitata* MBB | Ceske Budejovice, CZ.  48.976784, 14.452755 | OR670387 | Free-living: freshwater |  |
| *Trepomonas* *latecapitata* SZGHO | Schwarzenbruck, DE.  49.351984, 11.222120 | OR670388 | Free-living: freshwater |  |
| *Trepomonas* *latecapitata* SGR | Ezelsdorf, DE.  49.329915, 11.335027 | OR670389 | Free-living: freshwater |  |
| *Trepomonas* *latecapitata* CZ001 | Dubné, CZ.  48.969131, 14.372647 | OR670390 | Free-living: freshwater |  |
| *Trepomonas* *latecapitata* FE666 | Fucking (currently Fugging), AT.  48.061624, 12.858508 | OR670391 | Free-living: freshwater |  |
| *Trepomonas* *latecapitata* SEHP | Ceske Budejovice, CZ.  48.976784, 14.452755 | OR670392 | Free-living: freshwater |  |
| *Trepomonas* *steinii* Volt | Voltera, IT.  43.452889, 10.799752 | OR670406 | Free-living: freshwater |  |
| *Trepomonas* *steinii* 77MVwsc | Marsons Mills, MA, USA.  41.646640, -70.408458 | OR670407 | Free-living: marine |  |
| *Trepomonas* *steinii* CONGO | Mazancová et al. 2023 | OR670377 | Free-living: freshwater |  |
| *Trepomonas* *rotans* Volt | Voltera, IT.  43.452889, 10.799752 | OR670408 | Free-living: freshwater |  |
| *Trepomonas* *agilis* FE | Fucking (currently Fugging), AT.  48.061624, 12.858508 | OR670409 | Free-living: freshwater |  |
| *Trepomonas* *agilis* SZGHO | Schwarzenbruck, DE.  49.351984, 11.222120 | OR670410 | Sewage treatment facility: freshwater |  |
| *Trepomonas* *agilis* ZMB3 | Close to Mongu, ZM.  -15.4682711, 23.2893108 | OR670411 | Free-living: freshwater |  |
| *Trepomonas* *agilis* Pig | Sanpong village, TH | OR670378 | Pig feces |  |
| *Trepomonas* *agilis* MIS2C | Mazancová et al. 2023 | OR670379 | Free-living: freshwater |  |
| *Trepomonas* *agilis* SOOS4 | Mazancová et al. 2023 | OR670380 | Free-living: freshwater |  |
| *Trepomonas* sp. PPS6 Schön | Schönenburg, DE.  49.506886, 11.307145 | OR670393 | Free-living: freshwater |  |
| *Trepomonas* sp. PPS6 ZMB2 | Close to Sioma, ZM, Africa.  -16.599075, 23.516286 | OR670394 | Free-living: freshwater |  |
| *Trepomonas* sp. PPS6 Chat | Northwest Harbor, Long Island, NY, USA.  40.990546, -72.242103 | OR670395 | Free-living: freshwater |  |
| *Trepomonas* sp. PPS6 OD | Amagansett, Long Island, NY, USA.  40.9668306, -72.1358610 | OR670396 | Free-living: freshwater |  |
| *Trepomonas* sp. PPS6 BOT | Prague, CZ.  50.043884, 14.267198 | OR670372 | Free-living: freshwater |  |
| *Trepomonas* sp. PPS6 MACHUPICCHU | Mazancová et al. 2023 | OR670373 | Free-living: freshwater |  |
| *Trepomonas* sp. PPS6 VLADA7 | Mazancová et al. 2023 | OR670374 | Free-living: freshwater |  |
| *Trepomonas* sp. PPS6 DAR | Mazancová et al. 2023 | OR670375 | Free-living: freshwater |  |
| *Trepomonas* sp. PPS6 ERAWAN | Mazancová et al. 2023 | OR670376 | Free-living: freshwater |  |
| *Trepomonas* sp. PPS6 SM | Schwarzenbach Moore, DE.  49.355350, 11.229707 | OR670397 | Free-living: freshwater |  |
| *Trepomonas* sp. PPS6 ZMB5 | Close to Watopa, ZM, Africa.  -14.042405, 23.709317 | OR670398 | Free-living: freshwater |  |
| *Trepomonas* sp. PPS6 NO666 | Svalbard, NO.  78.639336, 16.732286 | OR670399 | Free-living: freshwater |  |
| *Trepomonas* sp. PPS6 EricRI | Charlestown, RI, USA.  41.364039, -71.654013 | OR670399 | Free-living: marine |  |
| *Trepomonas* sp. PPS6 PC002 | Prince Cove, Marstons Mills, MA, USA.  41.641629, -70.413456 | OR670401 | Free-living: marine |  |
| *Trepomonas* sp. PPS6 77MVTP | Marstons Mills, MA, USA.  41.646981, -70.406818 | OR670402 | Free-living: marine |  |
| *Trepomonas* sp. PPS6 77MVPH | Marstons Mills, MA, USA.  41.647230, -70.406076 | OR670403 | Free-living: marine |  |
| *Trepomonas* sp. PPS6 MVwsc | Marstons Mills, MA, USA.  41.646640, -70.408458 | OR670404 | Free-living: marine |  |
| *Trepomonas* sp. Volt2666 | Voltera, IT.  43.452889, 10.799752 | OR670405 | Free-living: freshwater |  |
| *Gyromonas ambulans* SPINDL2 | Mazancová et al. 2023 | OR670384 | Free-living: freshwater |  |
| *Trimitus* sp. IT1 | Kolisko et al. 2008 | OR670382 |  |  |
| *Trimitus* sp. MPE232 | Marstons Mills, MA, USA.  41.650752, -70.414446 | OR670422 | Free-living: freshwater |  |
| *Trimitus* sp. MPE233 | Marstons Mills, MA, USA.  41.650752, -70.414446 | OR670419 | Free-living: freshwater |  |
| *Trimitus* sp. SZGHO | Schwarzenbruck, DE.  49.351984, 11.222120 | OR670420 | Sewage treatment facility: freshwater |  |
| *Trimitus* sp. AN | Schwarzenbruck, DE.  49.351984, 11.222120 | OR670421 | Sewage treatment facility: freshwater |  |
| *Trimitus* sp. FISH | Sanpong village, TH | OR670383 | Endobiotic: gut of Siluriformes catfish |  |
