# Supplementary material for: Expanded gene and taxon sampling of diplomonads shows multiple switches to parasitic and free-living lifestyle
Source: BMC Biol. 2024 Sep 27;22:217. doi: 10.1186/s12915-024-02013-w (PMC11437800; doi:10.1186/s12915-024-02013-w)

Maturase HydA

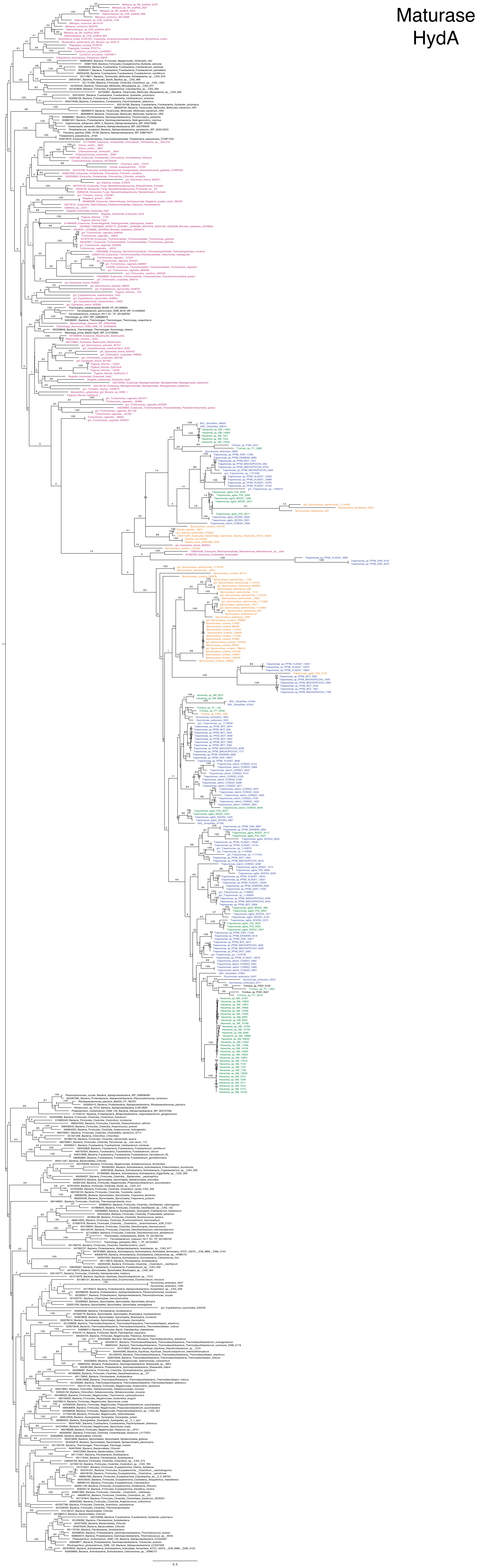

Maturase HydE

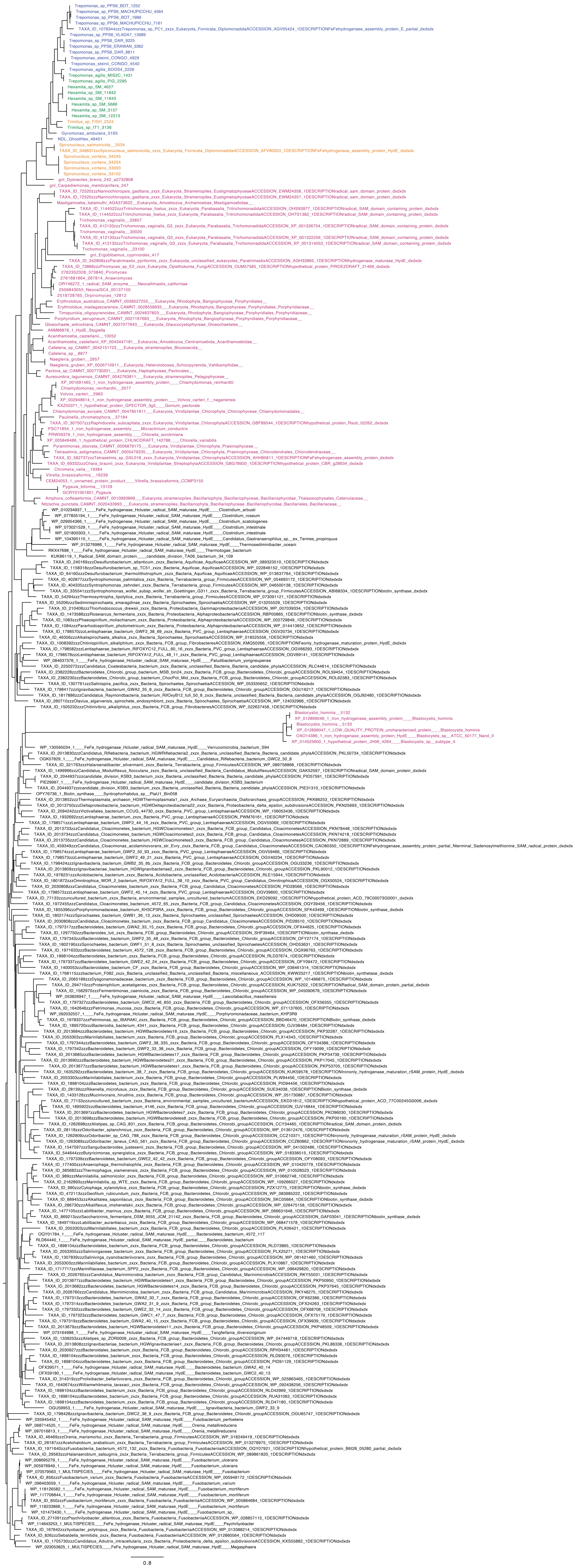

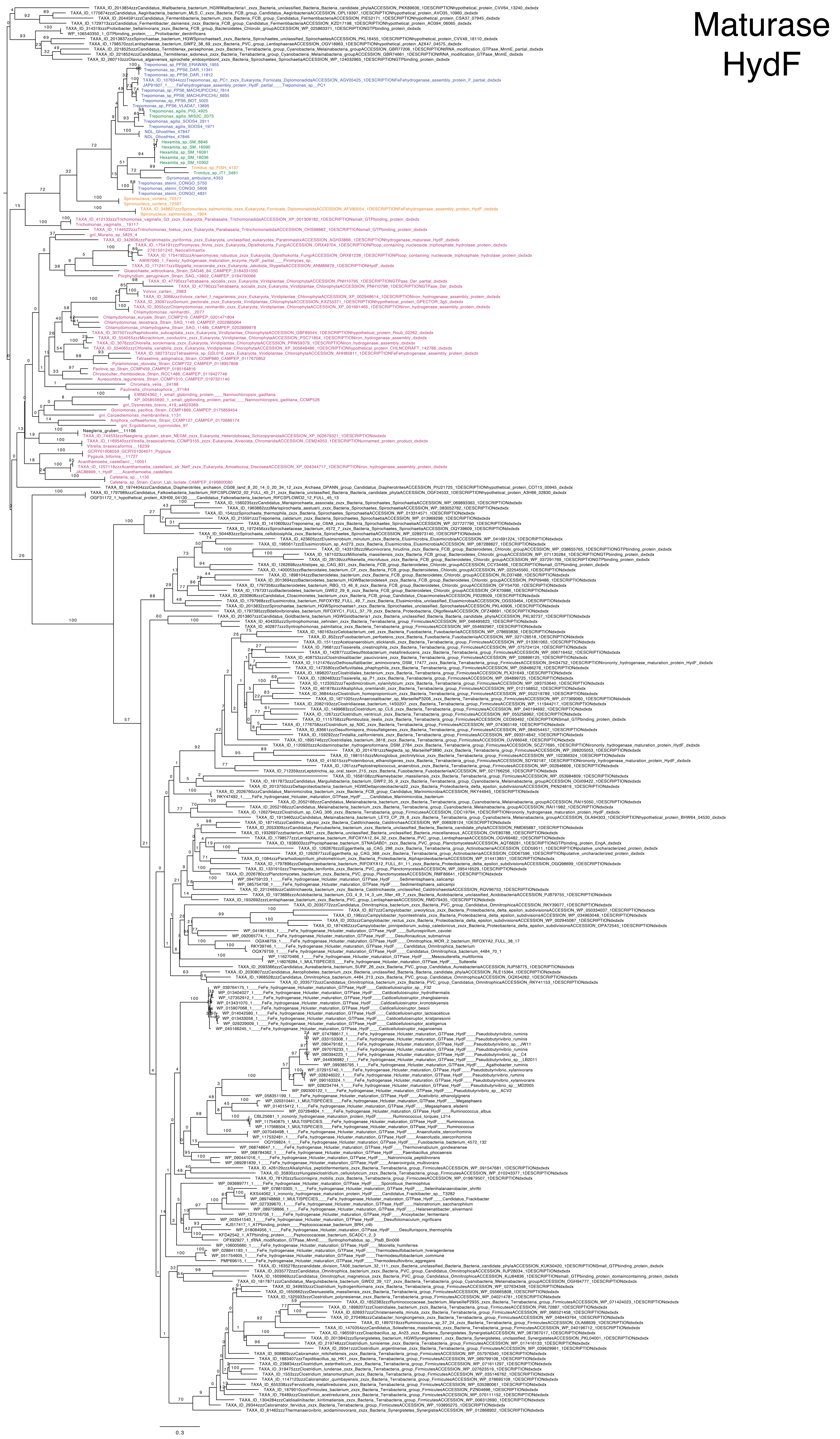
Maturase
HydF
0.3

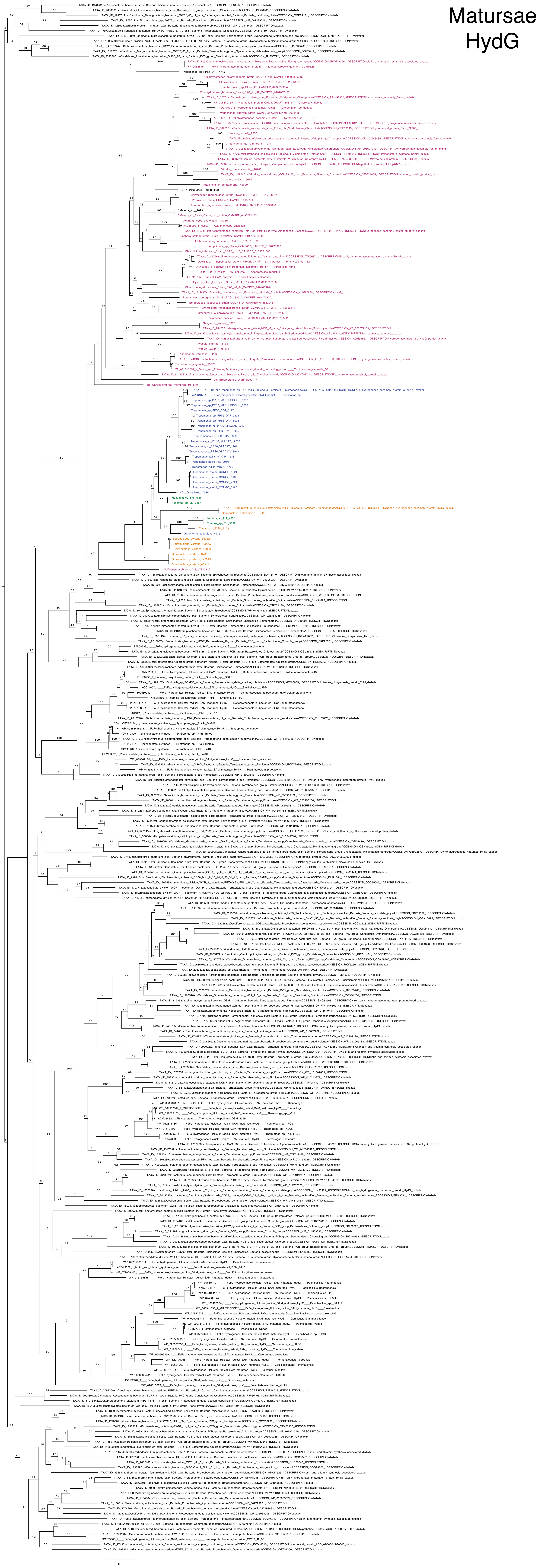
Matursae
HydG
0.2

Pyruvate:ferredoxin oxidoreductase (PFO)

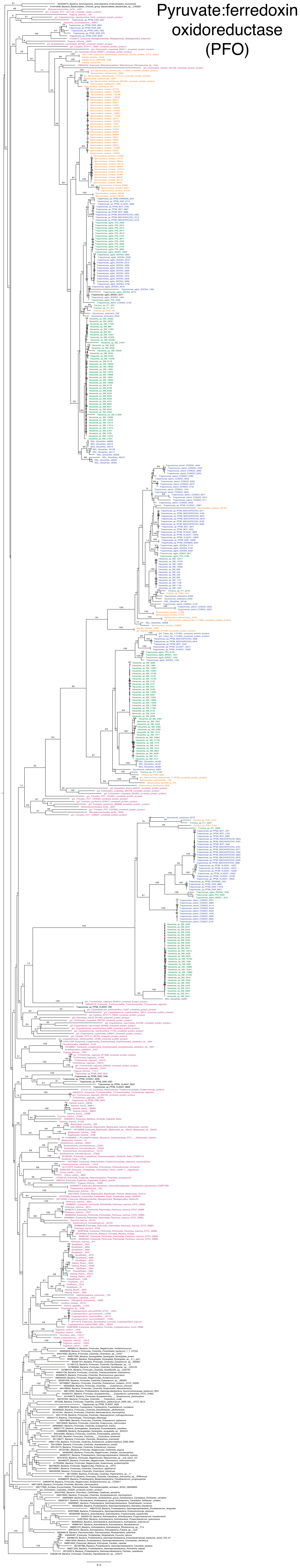

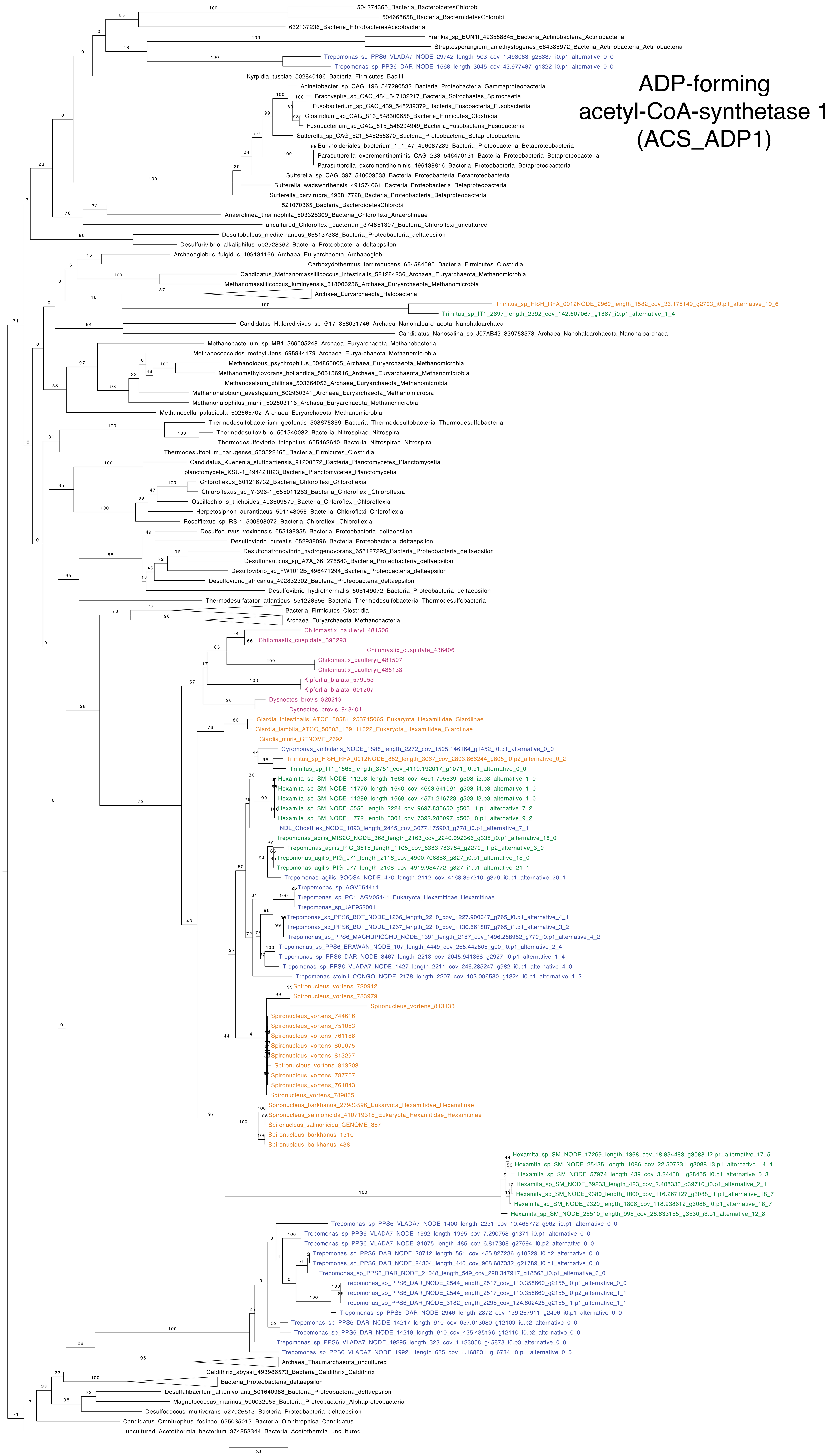
ADP-forming
acetyl-CoA-synthetase 1
(ACS_ADP1)
504374365_Bacteria_BacteroidetesChlorobi
504668658_Bacteria_BacteroidetesChlorobi
632137236_Bacteria_FibrobacteresAcidobacteria
Frankia_sp_EUN1f_493588845_Bacteria_Actinobacteria_Actinobacteria
Streptosporangium_amethystogenes_664388972_Bacteria_Actinobacteria_Actinobacteria
Trepomonas_sp_PPS6_VLADA7_NODE_29742_length_503_cov_1.493088_g26387_i0.p1_alternative_0_0
Trepomonas_sp_PPS6_DAR_NODE_1568_length_3045_cov_43.977487_g1322_i0.p1_alternative_0_0
Kyrpidia_tusciae_502840186_Bacteria_Firmicutes_Bacilli
Acinetobacter_sp_CAG_196_547290533_Bacteria_Proteobacteria_Gammaproteobacteria
Brachyspira_sp_CAG_484_547132217_Bacteria_Spirochaetes_Spirochaetia
Fusobacterium_sp_CAG_439_548239379_Bacteria_Fusobacteria_Fusobacteriia
Clostridium_sp_CAG_813_548300658_Bacteria_Firmicutes_Clostridia
Fusobacterium_sp_CAG_815_548294949_Bacteria_Fusobacteria_Fusobacteriia
Sutterella_sp_CAG_521_548255370_Bacteria_Proteobacteria_Betaproteobacteria
Burkholderiales_bacterium_1_1_47_496087239_Bacteria_Proteobacteria_Betaproteobacteria
Parasutterella_excrementihominis_CAG_233_546470131_Bacteria_Proteobacteria_Betaproteobacteria
Parasutterella_excrementihominis_496138816_Bacteria_Proteobacteria_Betaproteobacteria
Sutterella_sp_CAG_397_548009538_Bacteria_Proteobacteria_Betaproteobacteria
Sutterella_wadsworthensis_491574661_Bacteria_Proteobacteria_Betaproteobacteria
Sutterella_parvirubra_495817728_Bacteria_Proteobacteria_Betaproteobacteria
521070365_Bacteria_BacteroidetesChlorobi
Anaerolinea_thermophila_503325309_Bacteria_Chloroflexi_Anaerolineae
uncultured_Chloroflexi_bacterium_374851397_Bacteria_Chloroflexi_uncultured
Desulfobulbus_mediterraneus_655137388_Bacteria_Proteobacteria_deltaepsilon
Desulfurivibrio_alkaliphilus_502928362_Bacteria_Proteobacteria_deltaepsilon
Archaeoglobus_fulgidus_499181166_Archaea_Euryarchaeota_Archaeoglobi
Carboxydothermus_ferrireducens_654584596_Bacteria_Firmicutes_Clostridia
Candidatus_Methanomassiliicoccus_intestinalis_521284236_Archaea_Euryarchaeota_Methanomicrobia
Methanomassiliicoccus_luminyensis_518006236_Archaea_Euryarchaeota_Methanomicrobia
Archaea_Euryarchaeota_Halobacteria
Trimitus_sp_FISH_RFA_0012NODE_2969_length_1582_cov_33.175149_g2703_i0.p1_alternative_10_6
Trimitus_sp_IT1_2697_length_2392_cov_142.607067_g1867_i0.p1_alternative_1_4
Candidatus_Haloredivivus_sp_G17_358031746_Archaea_Nanohaloarchaeota_Nanohaloarchaea
Candidatus_Nanosalina_sp_J07AB43_339758578_Archaea_Nanohaloarchaeota_Nanohaloarchaea
Methanobacterium_sp_MB1_566005248_Archaea_Euryarchaeota_Methanobacteria
Methanococcoides_methylutens_695944179_Archaea_Euryarchaeota_Methanomicrobia
Methanolobus_psychrophilus_504866005_Archaea_Euryarchaeota_Methanomicrobia
Methanomethylovorans_hollandica_505136916_Archaea_Euryarchaeota_Methanomicrobia
Methanosalsum_zhilinae_503664056_Archaea_Euryarchaeota_Methanomicrobia
Methanohalobium_evestigatum_502960341_Archaea_Euryarchaeota_Methanomicrobia
Methanohalophilus_mahii_502803116_Archaea_Euryarchaeota_Methanomicrobia
Methanocella_paludicola_502665702_Archaea_Euryarchaeota_Methanomicrobia
Thermodesulfobacterium_geofontis_503675359_Bacteria_Thermodesulfobacteria_Thermodesulfobacteria
Thermodesulfovibrio_501540082_Bacteria_Nitrospirae_Nitrospira
Thermodesulfovibrio_thiophilus_655462640_Bacteria_Nitrospirae_Nitrospira
Thermodesulfobium_narugense_503522465_Bacteria_Firmicutes_Clostridia
Candidatus_Kuenenia_stuttgartiensis_91200872_Bacteria_Planctomycetes_Planctomycetia
planctomycete_KSU-1_494421823_Bacteria_Planctomycetes_Planctomycetia
Chloroflexus_501216732_Bacteria_Chloroflexi_Chloroflexia
Chloroflexus_sp_Y-396-1_655011263_Bacteria_Chloroflexi_Chloroflexia
Oscillochloris_trichoides_493609570_Bacteria_Chloroflexi_Chloroflexia
Herpetosiphon_aurantiacus_501143055_Bacteria_Chloroflexi_Chloroflexia
Roseiflexus_sp_RS-1_500598072_Bacteria_Chloroflexi_Chloroflexia
Desulfocurvus_vexinensis_655139355_Bacteria_Proteobacteria_deltaepsilon
Desulfovibrio_putealis_652938096_Bacteria_Proteobacteria_deltaepsilon
Desulfonatronovibrio_hydrogenovorans_655127295_Bacteria_Proteobacteria_deltaepsilon
Desulfonauticus_sp_A7A_661275543_Bacteria_Proteobacteria_deltaepsilon
Desulfovibrio_sp_FW1012B_496471294_Bacteria_Proteobacteria_deltaepsilon
Desulfovibrio_africanus_492832302_Bacteria_Proteobacteria_deltaepsilon
Desulfovibrio_hydrothermalis_505149072_Bacteria_Proteobacteria_deltaepsilon
Thermodesulfatator_atlanticus_551228656_Bacteria_Thermodesulfobacteria_Thermodesulfobacteria
Bacteria_Firmicutes_Clostridia
Archaea_Euryarchaeota_Methanobacteria
Chilomastix_caulleryi_481506
Chilomastix_cuspidata_393293
Chilomastix_cuspidata_436406
Chilomastix_caulleryi_481507
Chilomastix_caulleryi_486133
Kipferlia_bialata_579953
Kipferlia_bialata_601207
Dysnectes_brevis_929219
Dysnectes_brevis_948404
Giardia_intestinalis_ATCC_50581_253745065_Eukaryota_Hexamitidae_Giardiinae
Giardia_lamblia_ATCC_50803_159111022_Eukaryota_Hexamitidae_Giardiinae
Giardia_muris_GENOME_2692
Gyromonas_ambulans_NODE_1888_length_2272_cov_1595.146164_g1452_i0.p1_alternative_0_0
Trimitus_sp_FISH_RFA_0012NODE_882_length_3067_cov_2803.866244_g805_i0.p2_alternative_0_2
Trimitus_sp_IT1_1565_length_3751_cov_4110.192017_g1071_i0.p1_alternative_0_0
Hexamita_sp_SM_NODE_11298_length_1668_cov_4691.795639_g503_i2.p3_alternative_1_0
Hexamita_sp_SM_NODE_11776_length_1640_cov_4663.641091_g503_i4.p3_alternative_1_0
Hexamita_sp_SM_NODE_11299_length_1668_cov_4571.246729_g503_i3.p3_alternative_1_0
Hexamita_sp_SM_NODE_5550_length_2224_cov_9697.836650_g503_i1.p1_alternative_7_2
Hexamita_sp_SM_NODE_1772_length_3304_cov_7392.285097_g503_i0.p1_alternative_9_2
NDL_GhostHex_NODE_1093_length_2445_cov_3077.175903_g778_i0.p1_alternative_7_1
Trepomonas_agilis_MIS2C_NODE_368_length_2163_cov_2240.092366_g335_i0.p1_alternative_18_0
Trepomonas_agilis_PIG_3615_length_1105_cov_6383.783784_g2279_i1.p2_alternative_3_0
Trepomonas_agilis_PIG_971_length_2116_cov_4900.706888_g827_i0.p1_alternative_18_0
Trepomonas_agilis_PIG_977_length_2108_cov_4919.934772_g827_i1.p1_alternative_21_1
Trepomonas_agilis_SOOS4_NODE_470_length_2112_cov_4168.897210_g379_i0.p1_alternative_20_1
Trepomonas_sp_AGV054411
Trepomonas_sp_PC1_AGV05441_Eukaryota_Hexamitidae_Hexamitinae
Trepomonas_sp_JAP952001
Trepomonas_sp_PPS6_BOT_NODE_1266_length_2210_cov_1227.900047_g765_i0.p1_alternative_4_1
Trepomonas_sp_PPS6_BOT_NODE_1267_length_2210_cov_1130.561887_g765_i1.p1_alternative_3_2
Trepomonas_sp_PPS6_MACHUPICCHU_NODE_1391_length_2187_cov_1496.288952_g779_i0.p1_alternative_4_2
Trepomonas_sp_PPS6_ERAWAN_NODE_107_length_4449_cov_268.442805_g90_i0.p1_alternative_2_4
Trepomonas_sp_PPS6_DAR_NODE_3467_length_2218_cov_2045.941368_g2927_i0.p1_alternative_1_4
Trepomonas_sp_PPS6_VLADA7_NODE_1427_length_2211_cov_246.285247_g982_i0.p1_alternative_4_0
Trepomonas_steinii_CONGO_NODE_2178_length_2207_cov_103.096580_g1824_i0.p1_alternative_1_3
Spironucleus_vortens_730912
Spironucleus_vortens_783979
Spironucleus_vortens_813133
Spironucleus_vortens_744616
Spironucleus_vortens_751053
Spironucleus_vortens_761188
Spironucleus_vortens_809075
Spironucleus_vortens_813297
Spironucleus_vortens_813203
Spironucleus_vortens_787767
Spironucleus_vortens_761843
Spironucleus_vortens_789855
Spironucleus_barkhanus_27983596_Eukaryota_Hexamitidae_Hexamitinae
Spironucleus_salmonicida_410719318_Eukaryota_Hexamitidae_Hexamitinae
Spironucleus_salmonicida_GENOME_857
Spironucleus_barkhanus_1310
Spironucleus_barkhanus_438
Hexamita_sp_SM_NODE_17269_length_1368_cov_18.834483_g3088_i2.p1_alternative_17_5
Hexamita_sp_SM_NODE_25435_length_1086_cov_22.507331_g3088_i3.p1_alternative_14_4
Hexamita_sp_SM_NODE_57974_length_439_cov_3.244681_g38455_i0.p1_alternative_0_3
Hexamita_sp_SM_NODE_59233_length_423_cov_2.408333_g39710_i0.p1_alternative_2_1
Hexamita_sp_SM_NODE_9380_length_1800_cov_116.267127_g3088_i1.p1_alternative_18_7
Hexamita_sp_SM_NODE_9320_length_1806_cov_118.938612_g3088_i0.p1_alternative_18_7
Hexamita_sp_SM_NODE_28510_length_998_cov_26.833155_g3530_i3.p1_alternative_12_8
Trepomonas_sp_PPS6_VLADA7_NODE_1400_length_2231_cov_10.465772_g962_i0.p1_alternative_0_0
Trepomonas_sp_PPS6_VLADA7_NODE_1992_length_1995_cov_7.290758_g1371_i0.p1_alternative_0_0
Trepomonas_sp_PPS6_VLADA7_NODE_31075_length_485_cov_6.817308_g27694_i0.p2_alternative_0_0
Trepomonas_sp_PPS6_DAR_NODE_20712_length_561_cov_455.827236_g18229_i0.p2_alternative_0_0
Trepomonas_sp_PPS6_DAR_NODE_24304_length_440_cov_968.687332_g21789_i0.p1_alternative_0_0
Trepomonas_sp_PPS6_DAR_NODE_21048_length_549_cov_298.347917_g18563_i0.p1_alternative_0_0
Trepomonas_sp_PPS6_DAR_NODE_2544_length_2517_cov_110.358660_g2155_i0.p1_alternative_0_0
Trepomonas_sp_PPS6_DAR_NODE_2544_length_2517_cov_110.358660_g2155_i0.p2_alternative_1_1
Trepomonas_sp_PPS6_DAR_NODE_3182_length_2296_cov_124.802425_g2155_i1.p1_alternative_1_1
Trepomonas_sp_PPS6_DAR_NODE_2946_length_2372_cov_139.267911_g2496_i0.p1_alternative_0_0
Trepomonas_sp_PPS6_DAR_NODE_14217_length_910_cov_657.013080_g12109_i0.p2_alternative_0_0
Trepomonas_sp_PPS6_DAR_NODE_14218_length_910_cov_425.435196_g12110_i0.p2_alternative_0_0
Trepomonas_sp_PPS6_VLADA7_NODE_49295_length_323_cov_1.133858_g45878_i0.p3_alternative_0_0
Trepomonas_sp_PPS6_VLADA7_NODE_19921_length_685_cov_1.168831_g16734_i0.p1_alternative_0_0
Archaea_Thaumarchaeota_uncultured
Caldithrix_abyssi_493986573_Bacteria_Caldithrix_Caldithrix
Bacteria_Proteobacteria_deltaepsilon
Desulfatibacillum_alkenivorans_501640988_Bacteria_Proteobacteria_deltaepsilon
Magnetococcus_marinus_500032055_Bacteria_Proteobacteria_Alphaproteobacteria
Desulfococcus_multivorans_527026513_Bacteria_Proteobacteria_deltaepsilon
Candidatus_Omnitrophus_fodinae_655035013_Bacteria_Omnitrophica_Candidatus
uncultured_Acetothermia_bacterium_374853344_Bacteria_Acetothermia_uncultured
0.3

ADP-forming acetyl-CoA-synthetase 2 (ACS_ADP2)

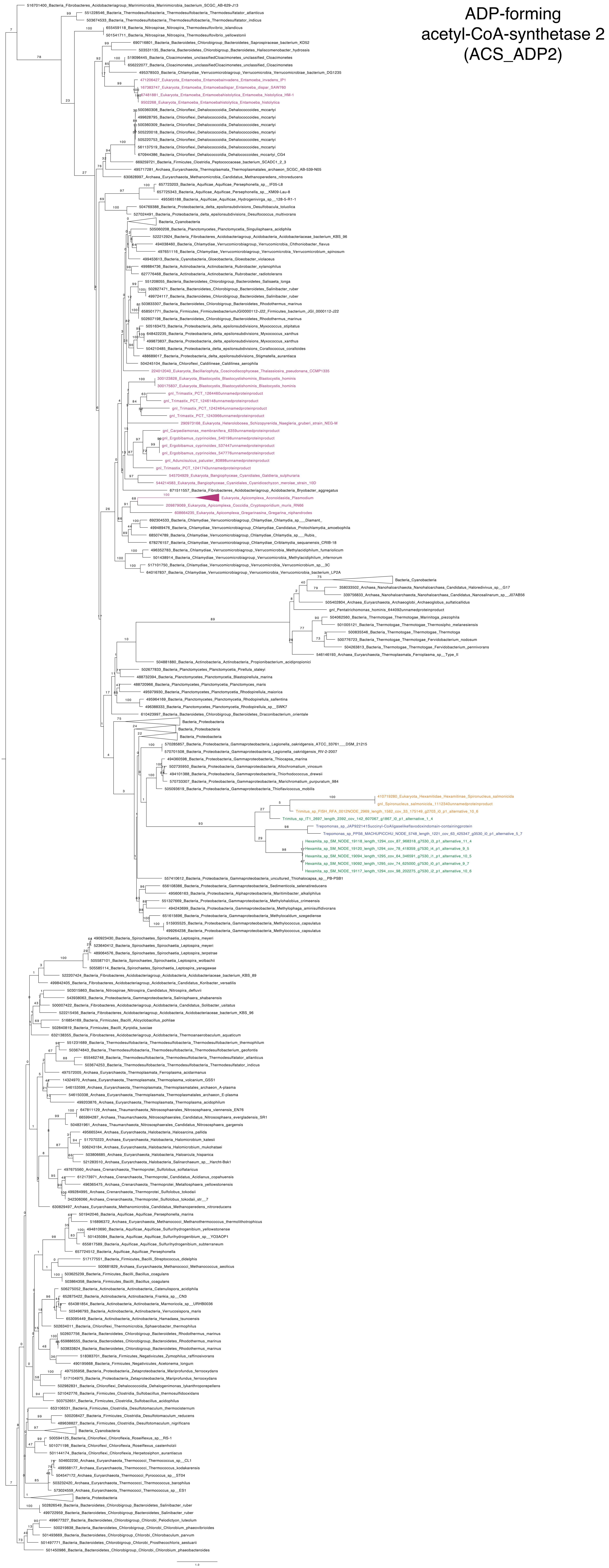

Supplement: Supplementary file 5 — Additional file 5: Fig. S2.Maximum Likelihood trees of the selected genes involved in energy generation and pyruvate metabolism. Trees were computed using IQ-TREE with C20+LG+G+F model, statistical support was inferred from 100 rapid bootstrap replicates computed in RAxML with LG4X+G model. Host associated diplomonads are marked in orange, free-living in blue, and green shows diplomonads whose lifestyle is uncertain. Other eukaryotes are colored in red. [file 12915_2024_2013_MOESM5_ESM.pdf]
