# Supplementary figures and images for: Expanded gene and taxon sampling of diplomonads shows multiple switches to parasitic and free-living lifestyle
Source: BMC Biol. 2024 Sep 27;22:217. doi: 10.1186/s12915-024-02013-w (PMC11437800; doi:10.1186/s12915-024-02013-w)

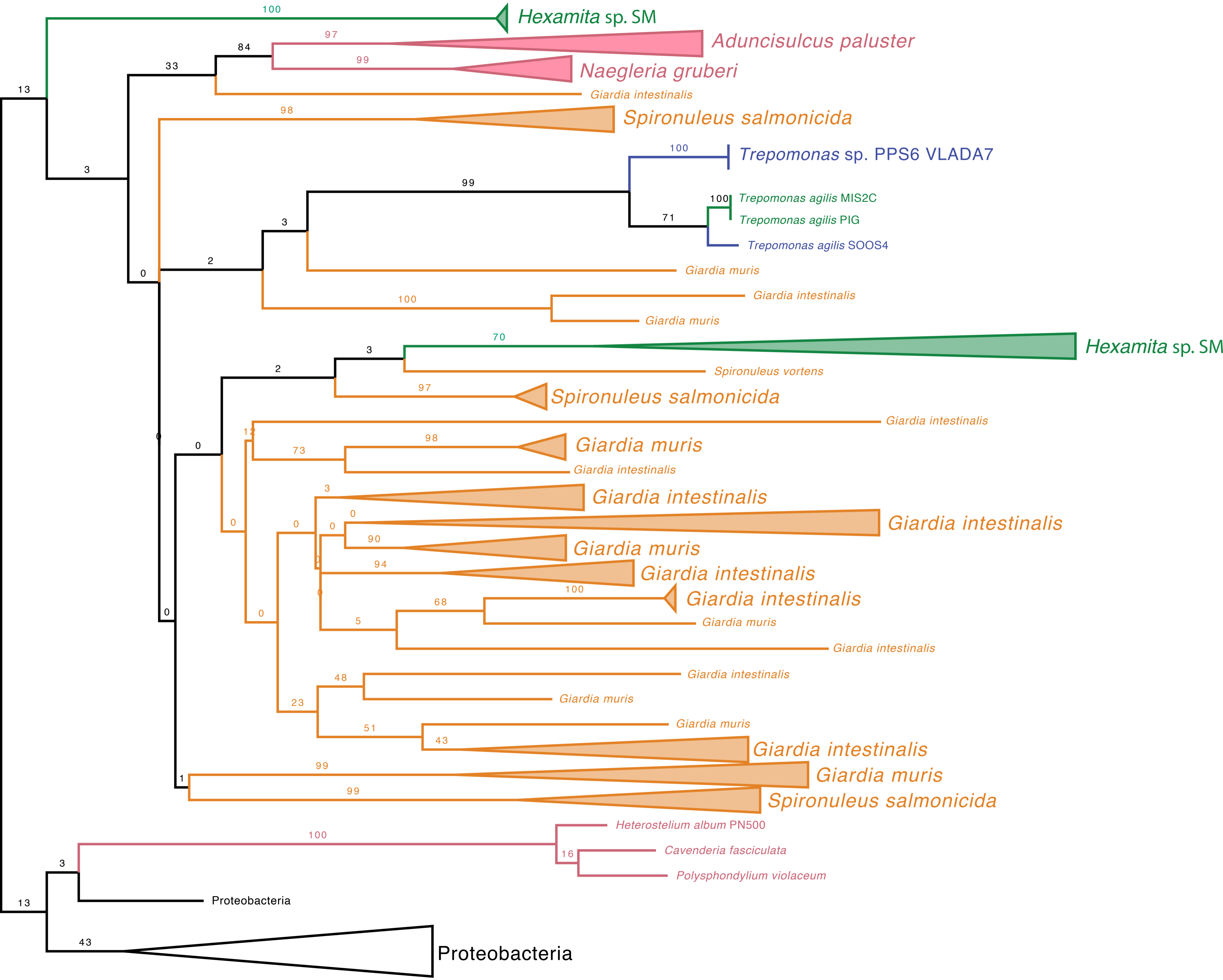

0.5

Supplement: Supplementary file 7 — Additional file 7: Fig. S4. Maximum likelihood tree of the tenascin gene. Tree was computed using RAxML with LG4X+G model and statistical support was inferred from 100 non-parametric bootstrap replicates. Host associated diplomonads are marked in orange, free-living in blue, and green shows diplomonads whose lifestyle is uncertain. Other eukaryotes are colored in red. [file 12915_2024_2013_MOESM7_ESM.pdf]

# BUSCO Assessment Results

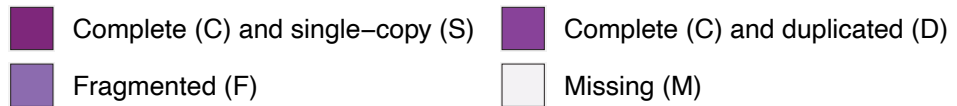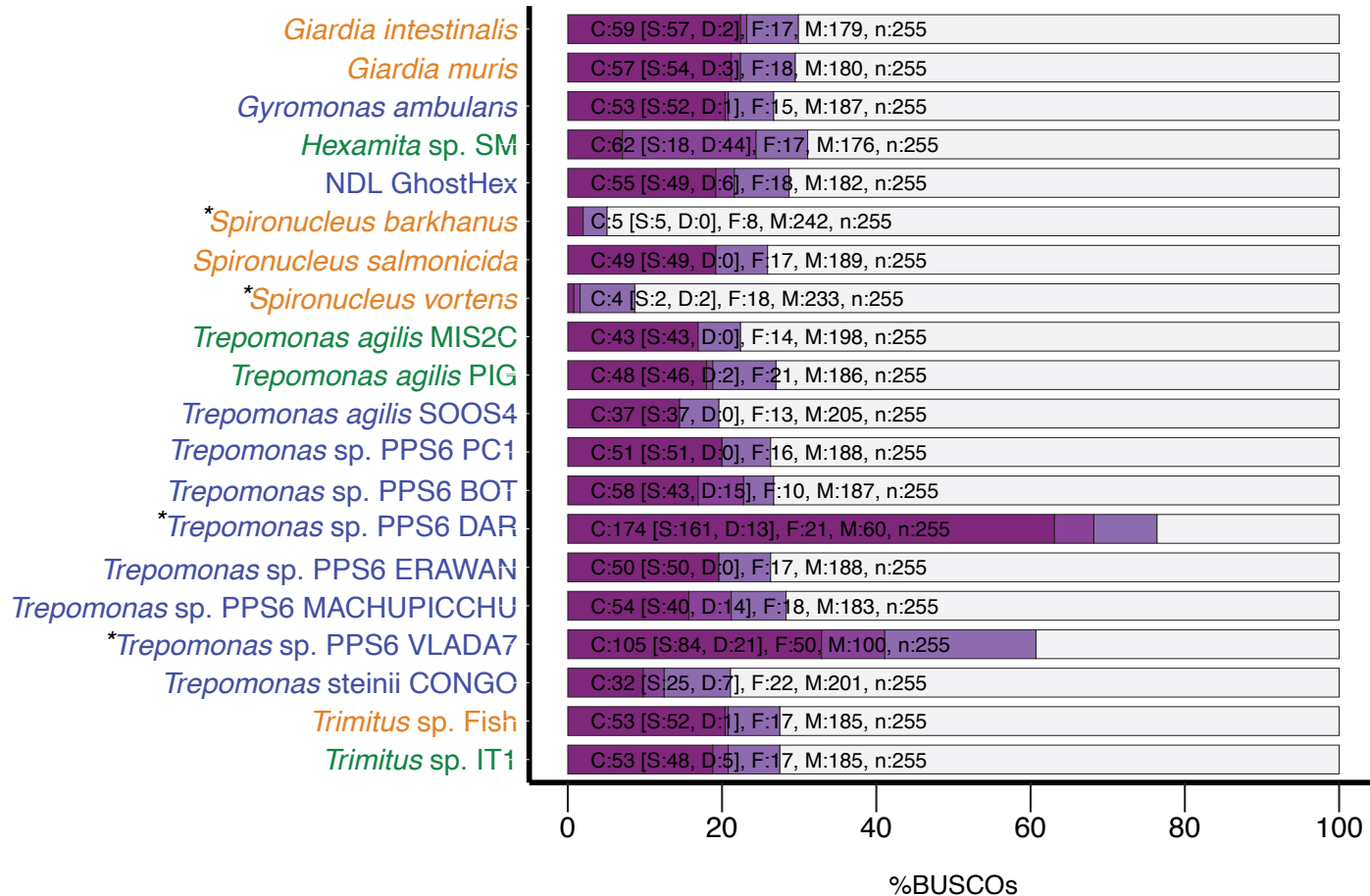

Supplement: Supplementary file 8 — Additional file 8: Fig. S5. Bar chart summarizing BUSCO results against eukaryotic lineages generated using generate_plot.py. An asterix in front of a species’s name denotes either incomplete (S. barkhanus, S. vortens) or contaminated (Trepomonas sp. PPS6 DAR, Trepomonas sp. PPS6 Vlada7) datasets. [file 12915_2024_2013_MOESM8_ESM.pdf]
