# Supplementary material for: Expanded gene and taxon sampling of diplomonads shows multiple switches to parasitic and free-living lifestyle
Source: BMC Biol. 2024 Sep 27;22:217. doi: 10.1186/s12915-024-02013-w (PMC11437800; doi:10.1186/s12915-024-02013-w)

Bactericidal Permeability Increasing-like protein (BPI)

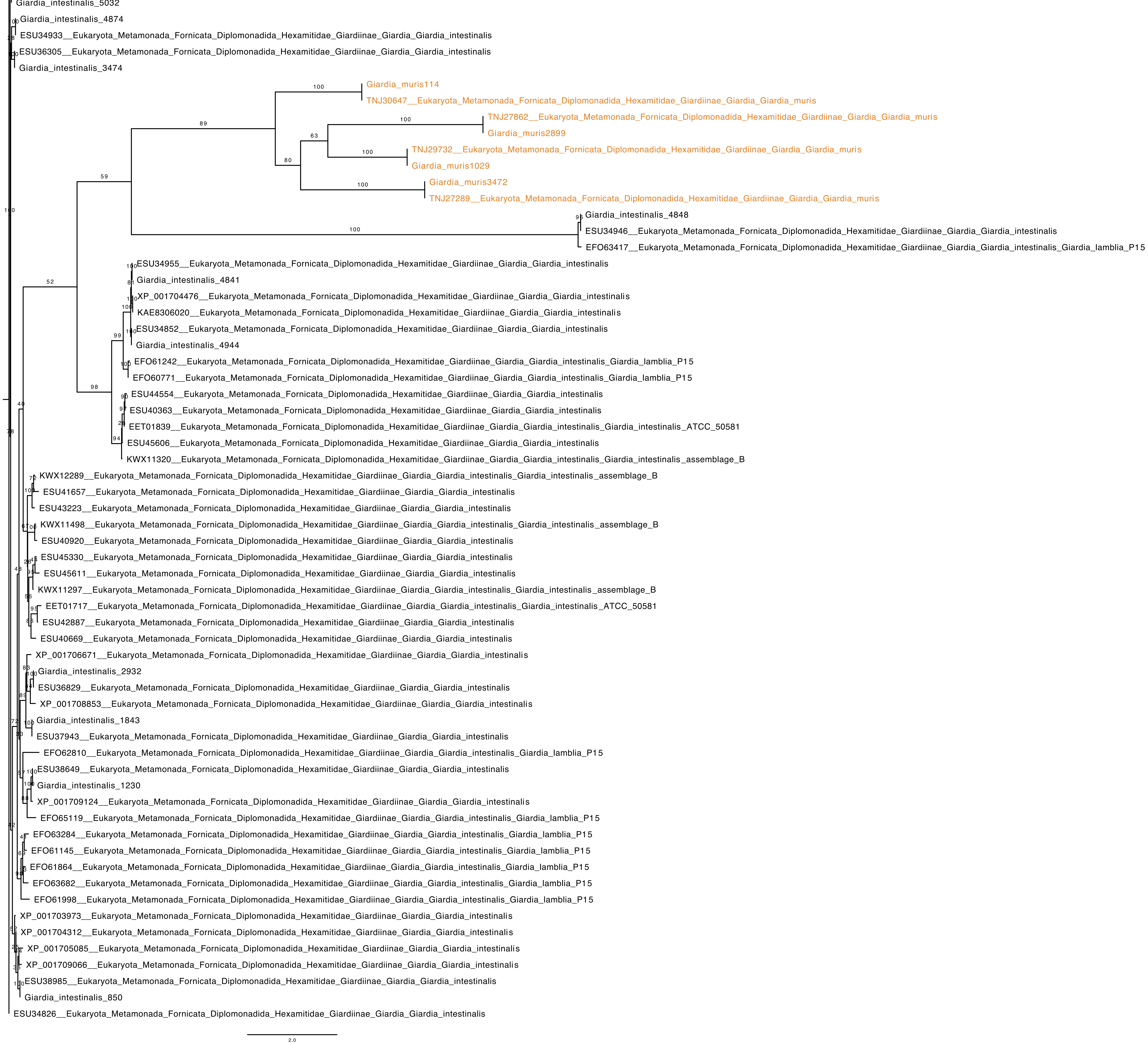

Cystatin

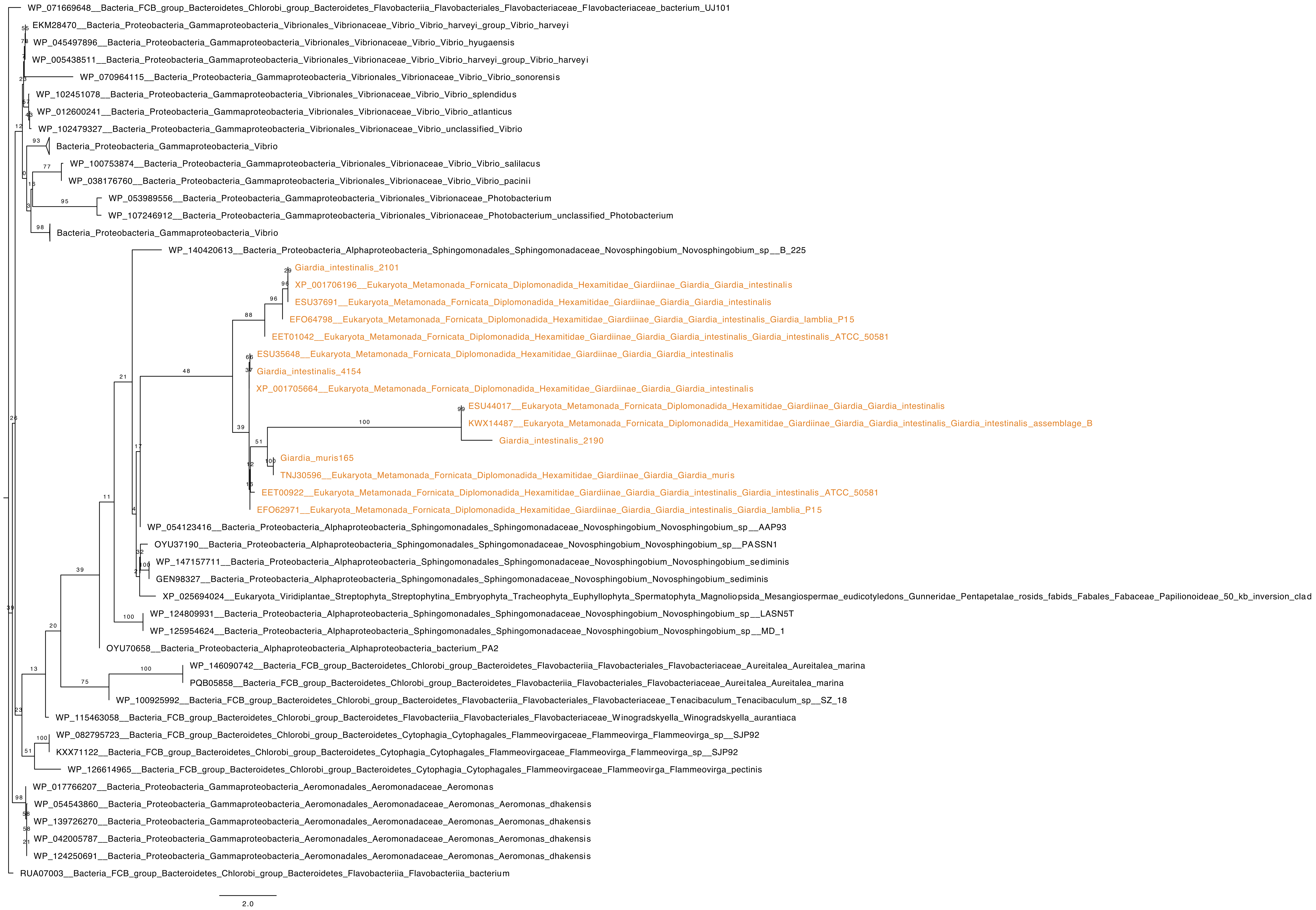
WP_071669648__Bacteria_FCB_group_Bacteroidetes_Chlorobi_group_Bacteroidetes_Flavobacteriia_Flavobacteriales_Flavobacteriaceae_Flavobacteriaceae_bacterium_UJ101
EKM28470__Bacteria_Proteobacteria_Gammaproteobacteria_Vibrionales_Vibrionaceae_Vibrio_Vibrio_harveyi_group_Vibrio_harveyi
WP_045497896__Bacteria_Proteobacteria_Gammaproteobacteria_Vibrionales_Vibrionaceae_Vibrio_Vibrio_hyugaensis
WP_005438511__Bacteria_Proteobacteria_Gammaproteobacteria_Vibrionales_Vibrionaceae_Vibrio_Vibrio_harveyi_group_Vibrio_harveyi
WP_070964115__Bacteria_Proteobacteria_Gammaproteobacteria_Vibrionales_Vibrionaceae_Vibrio_Vibrio_sonorensis
WP_102451078__Bacteria_Proteobacteria_Gammaproteobacteria_Vibrionales_Vibrionaceae_Vibrio_Vibrio_splendidus
WP_012600241__Bacteria_Proteobacteria_Gammaproteobacteria_Vibrionales_Vibrionaceae_Vibrio_Vibrio_atlanticus
WP_102479327__Bacteria_Proteobacteria_Gammaproteobacteria_Vibrionales_Vibrionaceae_Vibrio_unclassified_Vibrio
Bacteria_Proteobacteria_Gammaproteobacteria_Vibrio
WP_100753874__Bacteria_Proteobacteria_Gammaproteobacteria_Vibrionales_Vibrionaceae_Vibrio_Vibrio_salilacus
WP_038176760__Bacteria_Proteobacteria_Gammaproteobacteria_Vibrionales_Vibrionaceae_Vibrio_Vibrio_pacinii
WP_053989556__Bacteria_Proteobacteria_Gammaproteobacteria_Vibrionales_Vibrionaceae_Photobacterium
WP_107246912__Bacteria_Proteobacteria_Gammaproteobacteria_Vibrionales_Vibrionaceae_Photobacterium_unclassified_Photobacterium
Bacteria_Proteobacteria_Gammaproteobacteria_Vibrio
WP_140420613__Bacteria_Proteobacteria_Alphaproteobacteria_Sphingomonadales_Sphingomonadaceae_Novosphingobium_Novosphingobium_sp__B_225
Giardia_intestinalis_2101
XP_001706196__Eukaryota_Metamonada_Fornicata_Diplomonadida_Hexamitidae_Giardiinae_Giardia_Giardia_intestinalis
ESU37691__Eukaryota_Metamonada_Fornicata_Diplomonadida_Hexamitidae_Giardiinae_Giardia_Giardia_intestinalis
EFO64798__Eukaryota_Metamonada_Fornicata_Diplomonadida_Hexamitidae_Giardiinae_Giardia_Giardia_intestinalis_Giardia_lamblia_P15
EET01042__Eukaryota_Metamonada_Fornicata_Diplomonadida_Hexamitidae_Giardiinae_Giardia_Giardia_intestinalis_Giardia_intestinalis_ATCC_50581
ESU35648__Eukaryota_Metamonada_Fornicata_Diplomonadida_Hexamitidae_Giardiinae_Giardia_Giardia_intestinalis
Giardia_intestinalis_4154
XP_001705664__Eukaryota_Metamonada_Fornicata_Diplomonadida_Hexamitidae_Giardiinae_Giardia_Giardia_intestinalis
ESU44017__Eukaryota_Metamonada_Fornicata_Diplomonadida_Hexamitidae_Giardiinae_Giardia_Giardia_intestinalis
KWX14487__Eukaryota_Metamonada_Fornicata_Diplomonadida_Hexamitidae_Giardiinae_Giardia_Giardia_intestinalis_Giardia_intestinalis_assemblage_B
Giardia_intestinalis_2190
Giardia_muris165
TNJ30596__Eukaryota_Metamonada_Fornicata_Diplomonadida_Hexamitidae_Giardiinae_Giardia_Giardia_muris
EET00922__Eukaryota_Metamonada_Fornicata_Diplomonadida_Hexamitidae_Giardiinae_Giardia_Giardia_intestinalis_Giardia_intestinalis_ATCC_50581
EFO62971__Eukaryota_Metamonada_Fornicata_Diplomonadida_Hexamitidae_Giardiinae_Giardia_Giardia_intestinalis_Giardia_lamblia_P15
WP_054123416__Bacteria_Proteobacteria_Alphaproteobacteria_Sphingomonadales_Sphingomonadaceae_Novosphingobium_Novosphingobium_sp__AAP93
OYU37190__Bacteria_Proteobacteria_Alphaproteobacteria_Sphingomonadales_Sphingomonadaceae_Novosphingobium_Novosphingobium_sp__PASSN1
WP_147157711__Bacteria_Proteobacteria_Alphaproteobacteria_Sphingomonadales_Sphingomonadaceae_Novosphingobium_Novosphingobium_sediminis
GEN98327__Bacteria_Proteobacteria_Alphaproteobacteria_Sphingomonadales_Sphingomonadaceae_Novosphingobium_Novosphingobium_sediminis
XP_025694024__Eukaryota_Viridiplantae_Streptophyta_Streptophytina_Embryophyta_Tracheophyta_Euphyllophyta_Spermatophyta_Magnoliopsida_Mesangiospermae_eudicotyledons_Gunneridae_Pentapetalae_rosids_fabids_Fabales_Fabaceae_Papilionoideae_50_kb_inversion_clad
WP_124809931__Bacteria_Proteobacteria_Alphaproteobacteria_Sphingomonadales_Sphingomonadaceae_Novosphingobium_Novosphingobium_sp__LASN5T
WP_125954624__Bacteria_Proteobacteria_Alphaproteobacteria_Sphingomonadales_Sphingomonadaceae_Novosphingobium_Novosphingobium_sp__MD_1
OYU70658__Bacteria_Proteobacteria_Alphaproteobacteria_Alphaproteobacteria_bacterium_PA2
WP_146090742__Bacteria_FCB_group_Bacteroidetes_Chlorobi_group_Bacteroidetes_Flavobacteriia_Flavobacteriales_Flavobacteriaceae_Aureitalea_Aureitalea_marina
PQB05858__Bacteria_FCB_group_Bacteroidetes_Chlorobi_group_Bacteroidetes_Flavobacteriia_Flavobacteriales_Flavobacteriaceae_Aureitalea_Aureitalea_marina
WP_100925992__Bacteria_FCB_group_Bacteroidetes_Chlorobi_group_Bacteroidetes_Flavobacteriia_Flavobacteriales_Flavobacteriaceae_Tenacibaculum_Tenacibaculum_sp__SZ_18
WP_115463058__Bacteria_FCB_group_Bacteroidetes_Chlorobi_group_Bacteroidetes_Flavobacteriia_Flavobacteriales_Flavobacteriaceae_Winogradskyella_Winogradskyella_aurantiaca
WP_082795723__Bacteria_FCB_group_Bacteroidetes_Chlorobi_group_Bacteroidetes_Cytophagia_Cytophagales_Flammeovirgaceae_Flammeovirga_Flammeovirga_sp__SJP92
KXX71122__Bacteria_FCB_group_Bacteroidetes_Chlorobi_group_Bacteroidetes_Cytophagia_Cytophagales_Flammeovirgaceae_Flammeovirga_Flammeovirga_sp__SJP92
WP_126614965__Bacteria_FCB_group_Bacteroidetes_Chlorobi_group_Bacteroidetes_Cytophagia_Cytophagales_Flammeovirgaceae_Flammeovirga_Flammeovirga_pectinis
WP_017766207__Bacteria_Proteobacteria_Gammaproteobacteria_Aeromonadales_Aeromonadaceae_Aeromonas
WP_054543860__Bacteria_Proteobacteria_Gammaproteobacteria_Aeromonadales_Aeromonadaceae_Aeromonas_Aeromonas_dhakensis
WP_139726270__Bacteria_Proteobacteria_Gammaproteobacteria_Aeromonadales_Aeromonadaceae_Aeromonas_Aeromonas_dhakensis
WP_042005787__Bacteria_Proteobacteria_Gammaproteobacteria_Aeromonadales_Aeromonadaceae_Aeromonas_Aeromonas_dhakensis
WP_124250691__Bacteria_Proteobacteria_Gammaproteobacteria_Aeromonadales_Aeromonadaceae_Aeromonas_Aeromonas_dhakensis
RUA07003__Bacteria_FCB_group_Bacteroidetes_Chlorobi_group_Bacteroidetes_Flavobacteriia_Flavobacteriia_bacterium
2.0

Fructose bisphosphate-aldolase (FBPA)

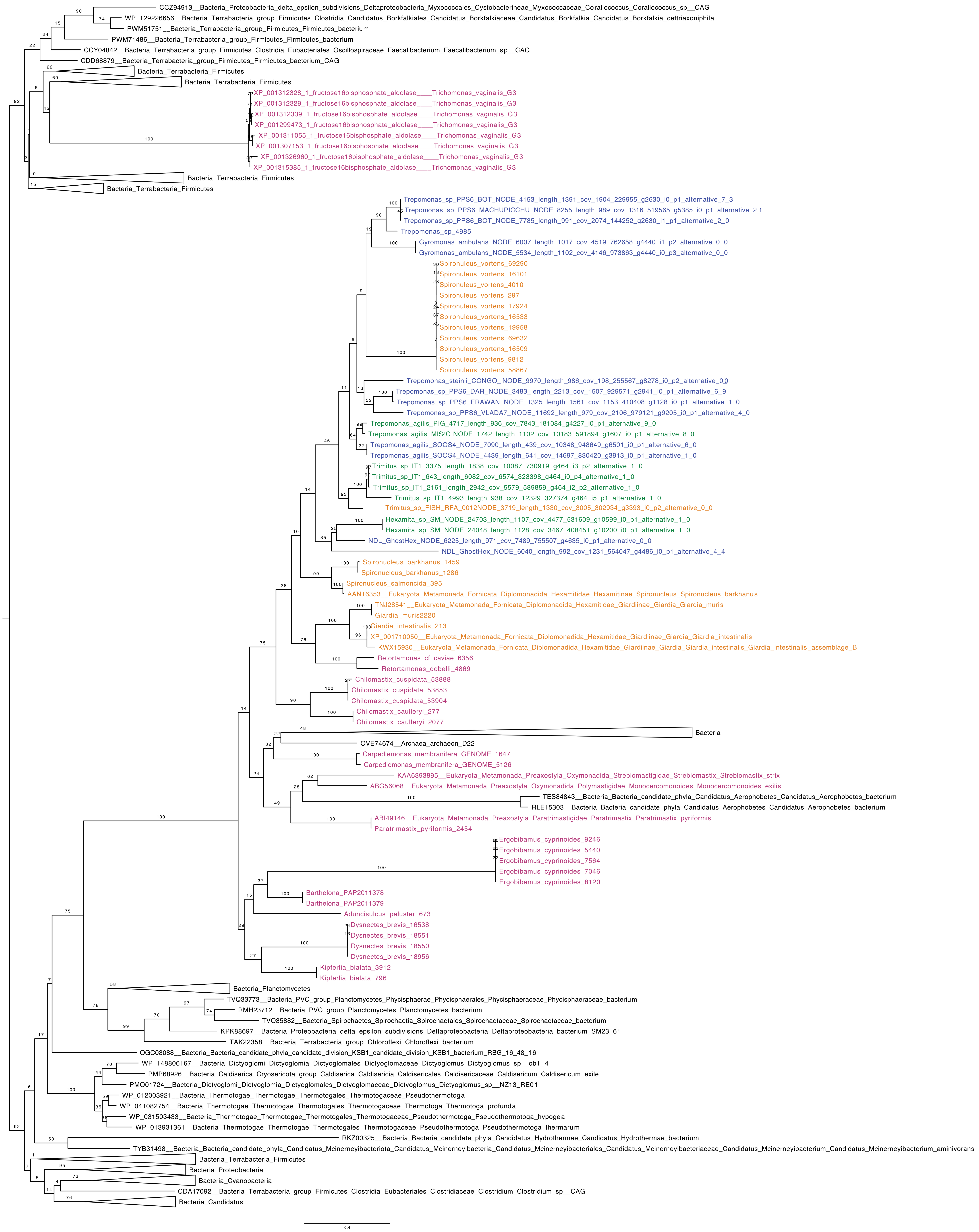

Enolase

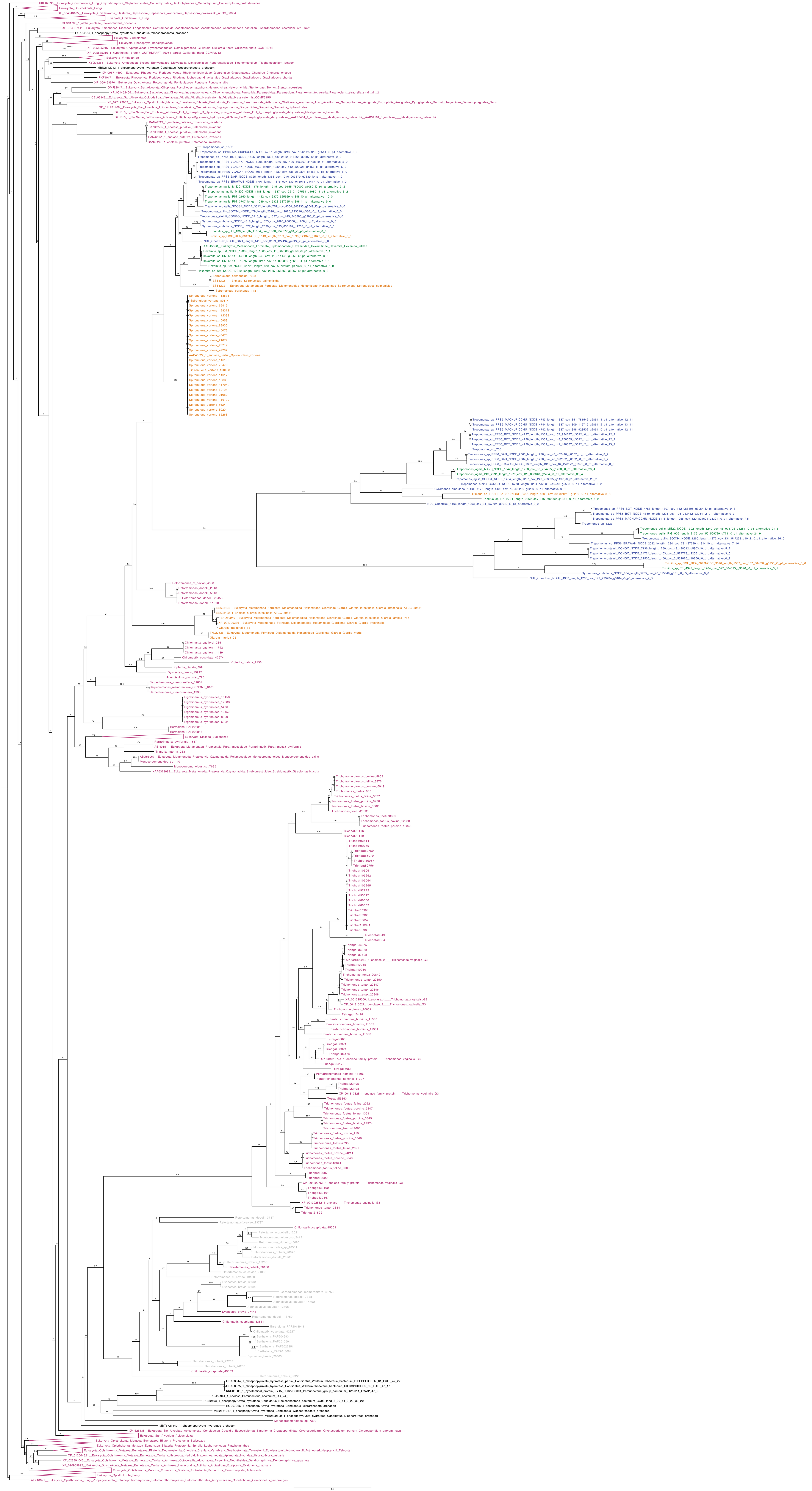

Arginine deiminase (ADI)

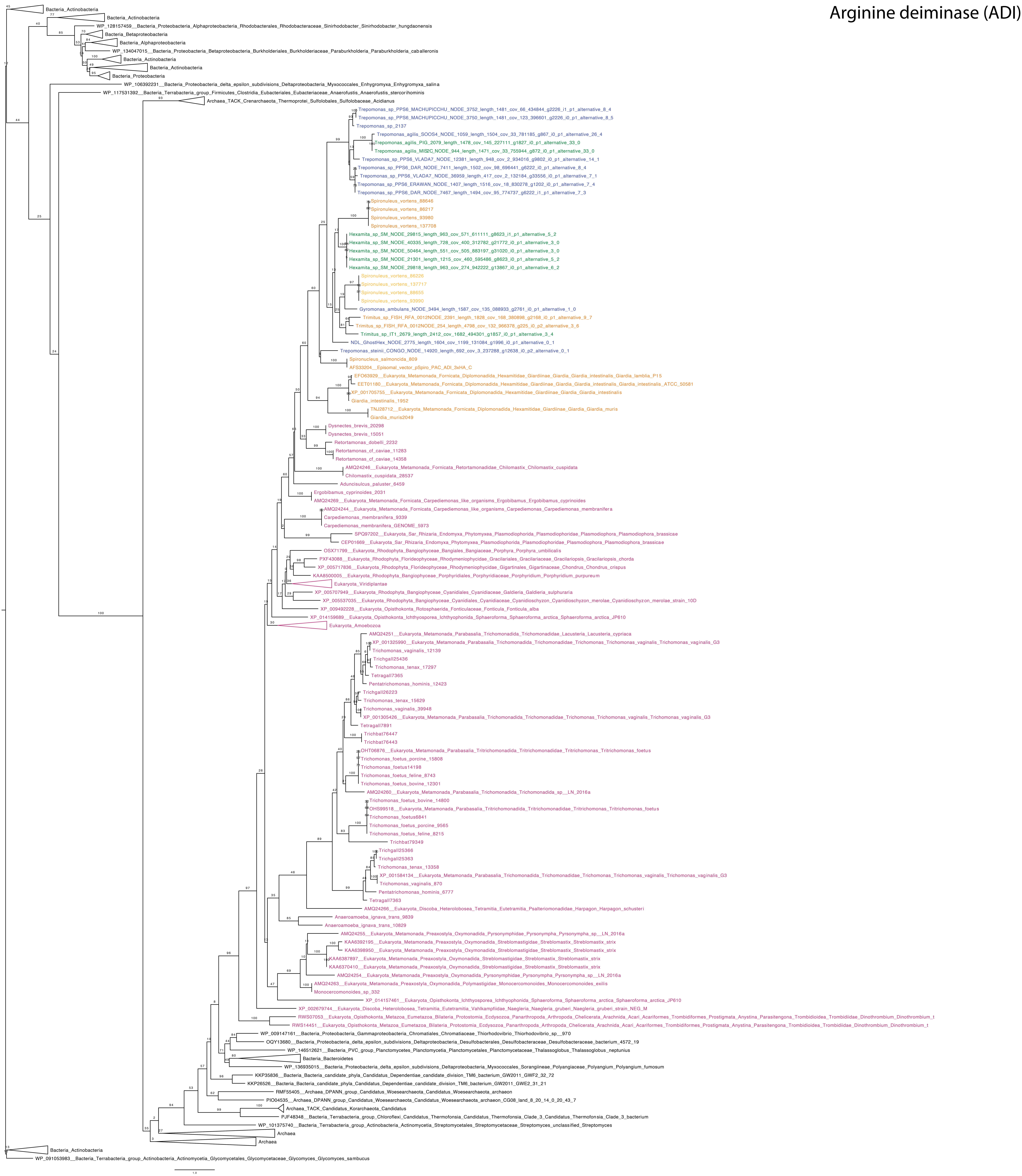

pyridoxamine 5′-phosphate osidase (PNPO)

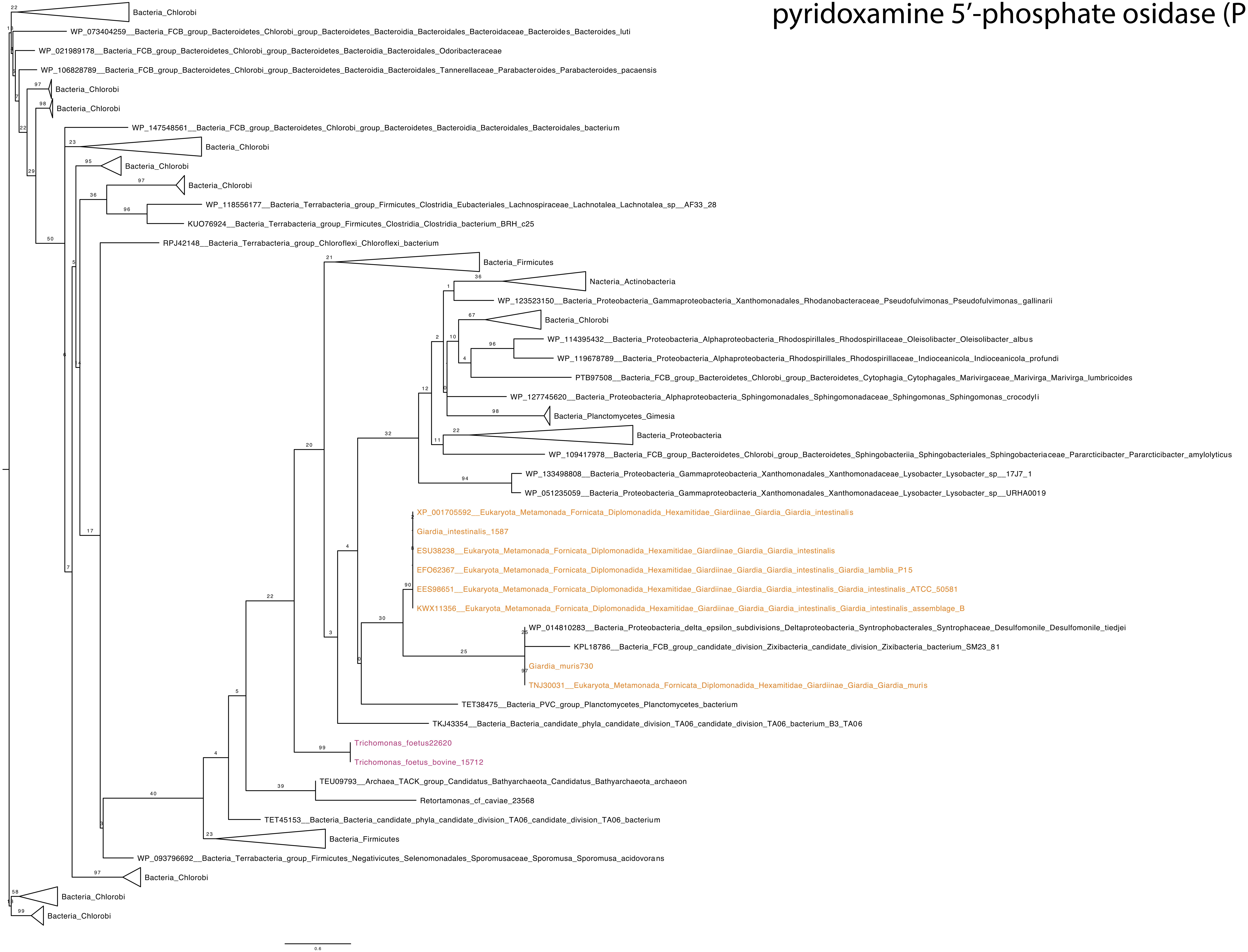

Ornithine transcarbamylase (OTC)

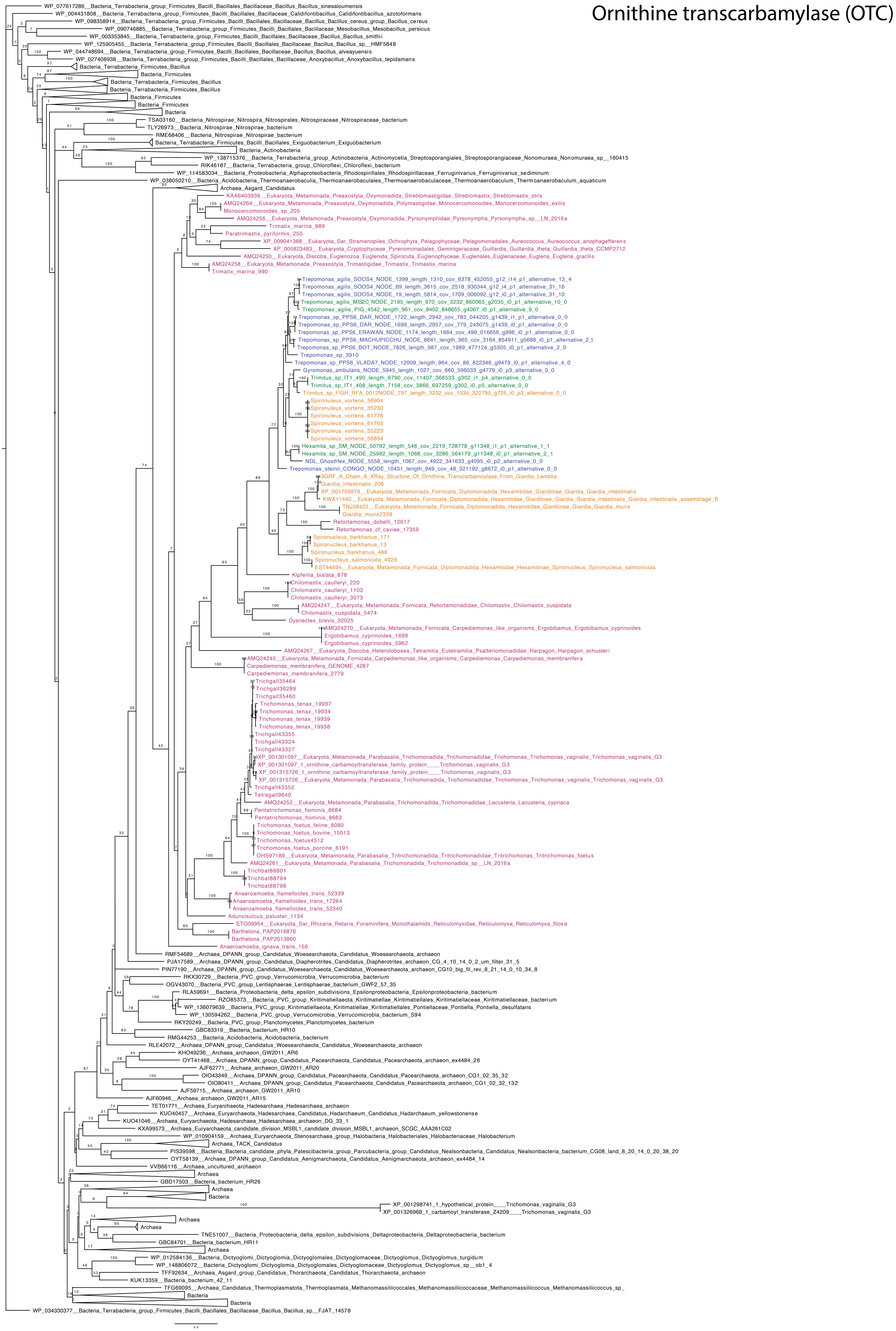

NADP specific glutamate dehydrogenase (NADP-GDH)

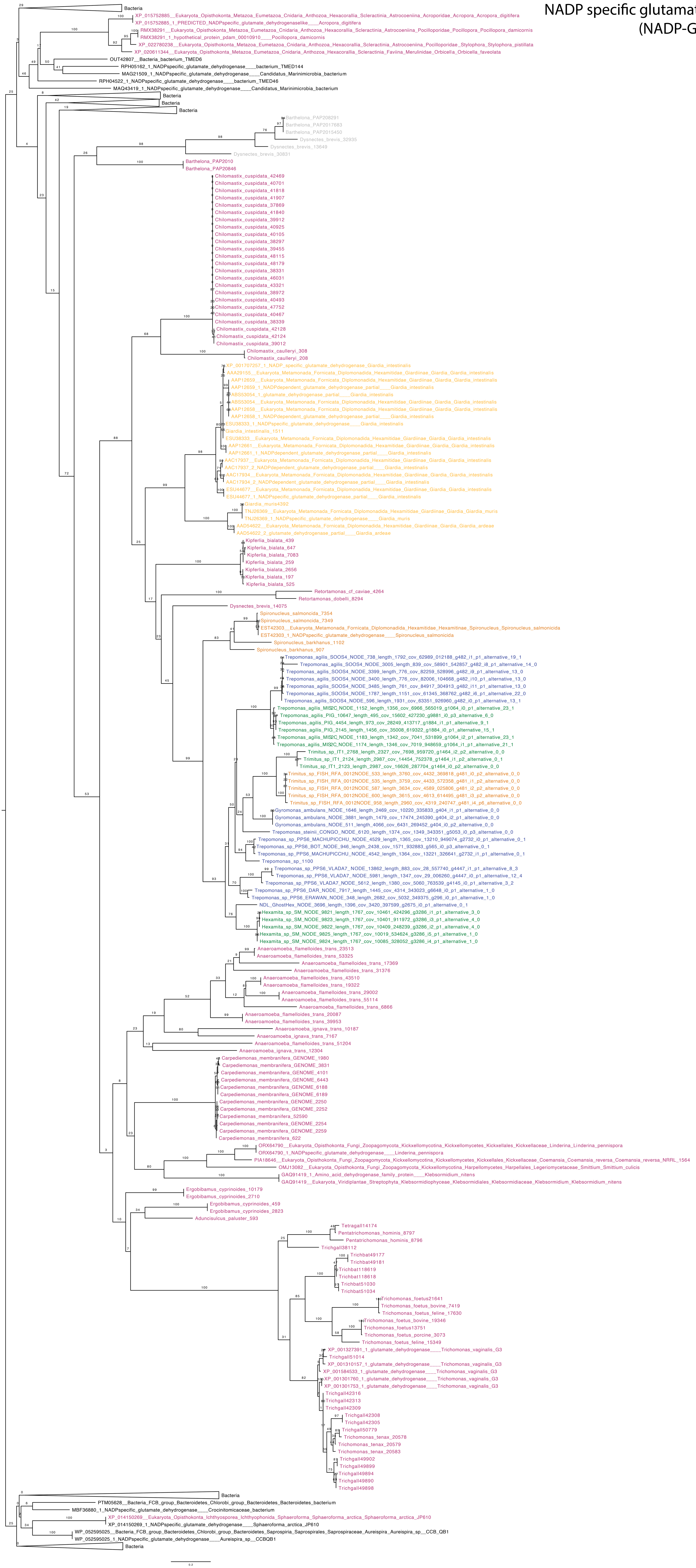

Extracellular nuclease

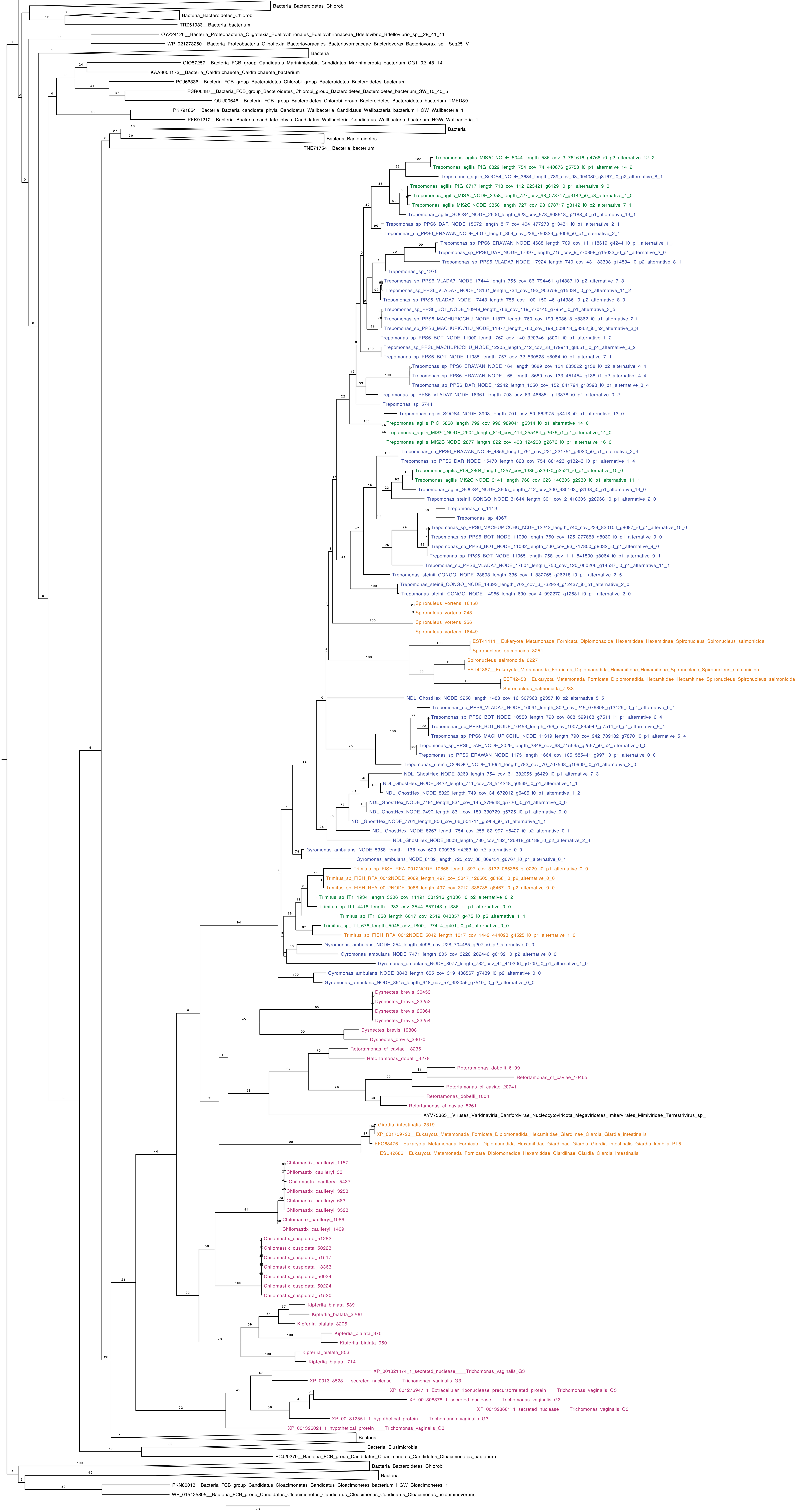

Cathepsin B

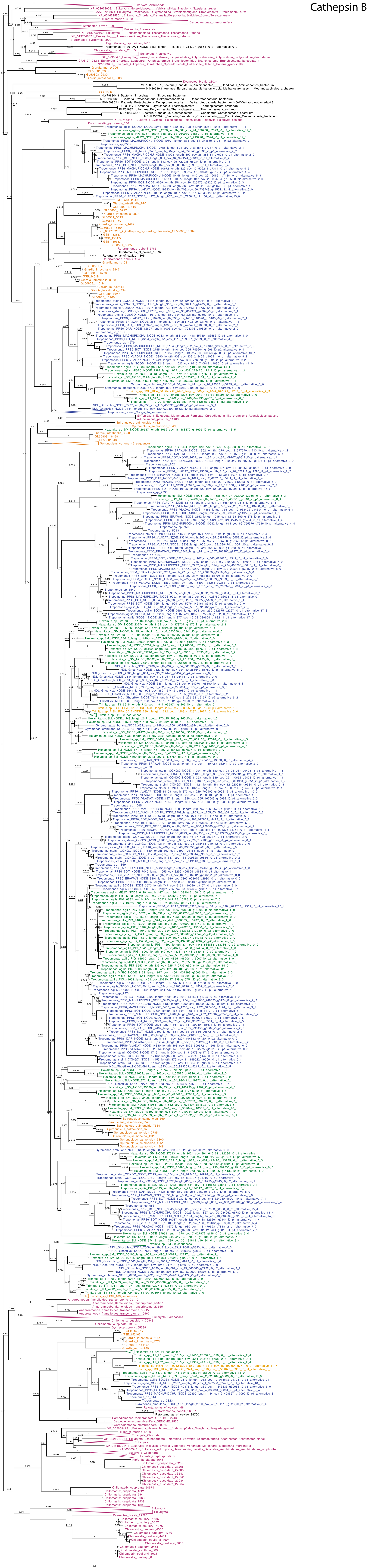

Thioredoxin

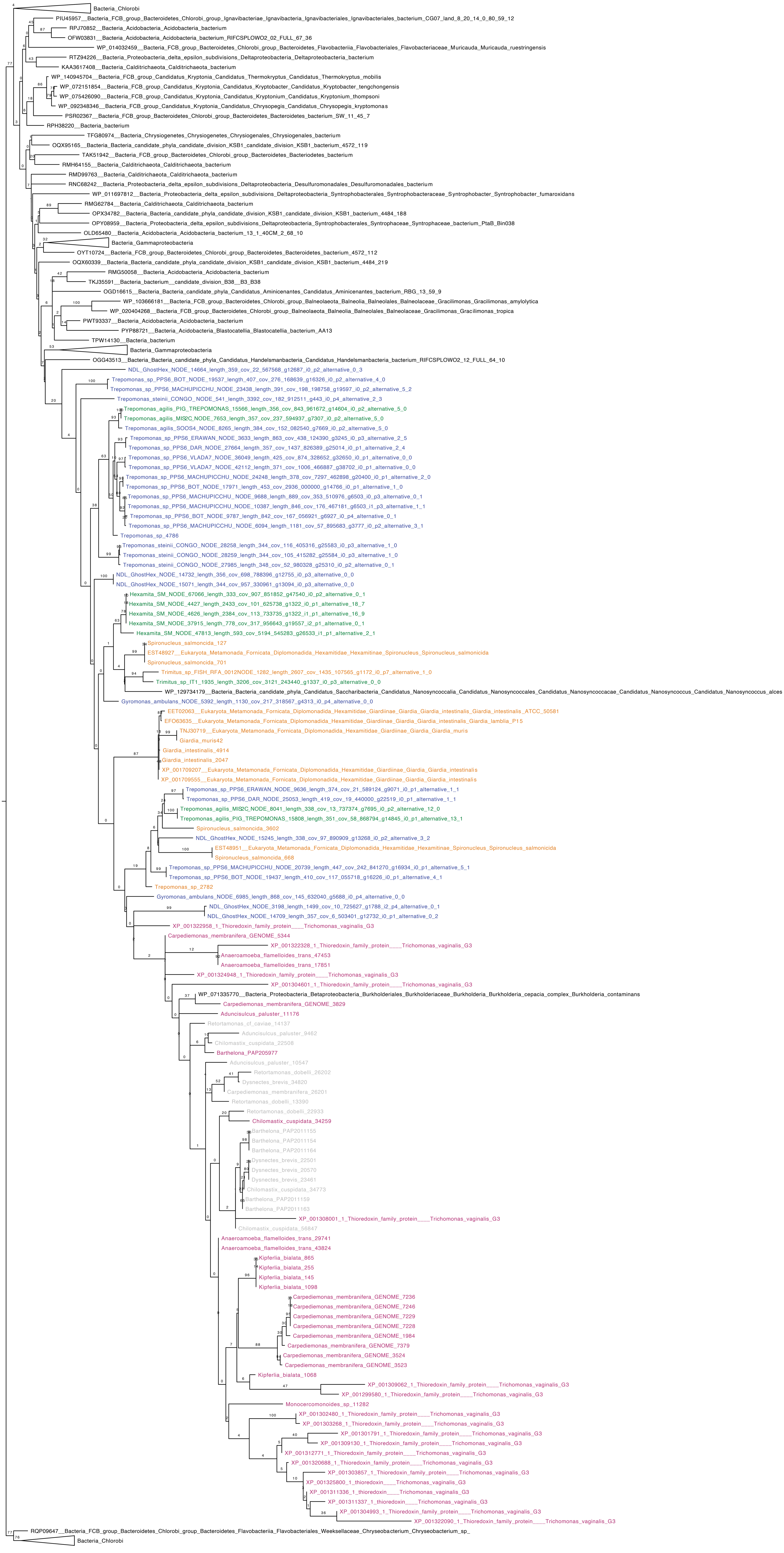

Tenascin

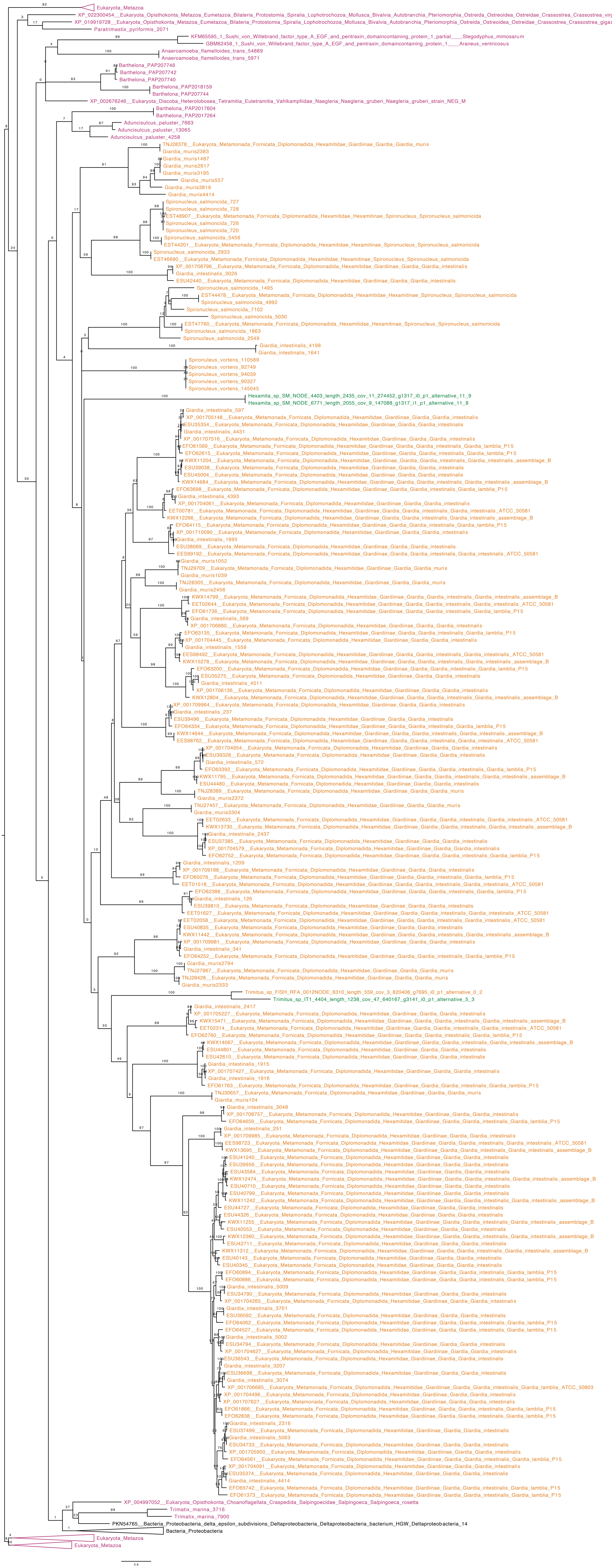

Supplement: Supplementary file 3 — Additional file 3: Fig. S1. Maximum Likelihood trees showing selected virulence factors. IQ-TREE with C20+LG+G+F model, statistical support was inferred from 100 rapid bootstrap replicates computed in RAxML with LG4X+G model. Host associated diplomonads are marked in orange, free-living in blue, and green shows diplomonads whose lifestyle is uncertain. Other eukaryotes are colored in red. To improve readability of the trees, large bacterial or eukaryotic clades were collapsed. Sequences that are possible bacterial contamination were marked in grey. [file 12915_2024_2013_MOESM3_ESM.pdf]
