# Supplementary material for: Expanded gene and taxon sampling of diplomonads shows multiple switches to parasitic and free-living lifestyle
Source: BMC Biol. 2024 Sep 27;22:217. doi: 10.1186/s12915-024-02013-w (PMC11437800; doi:10.1186/s12915-024-02013-w)

Lysozyme

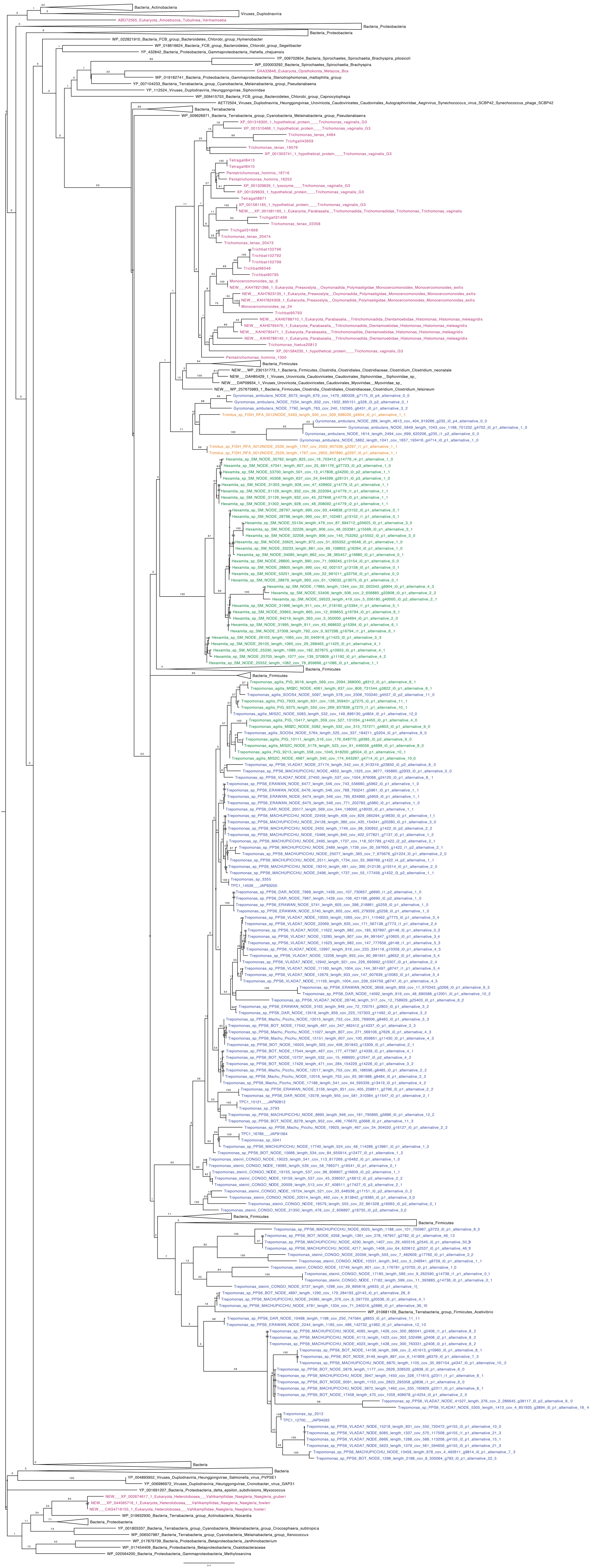

Murein hydrolase

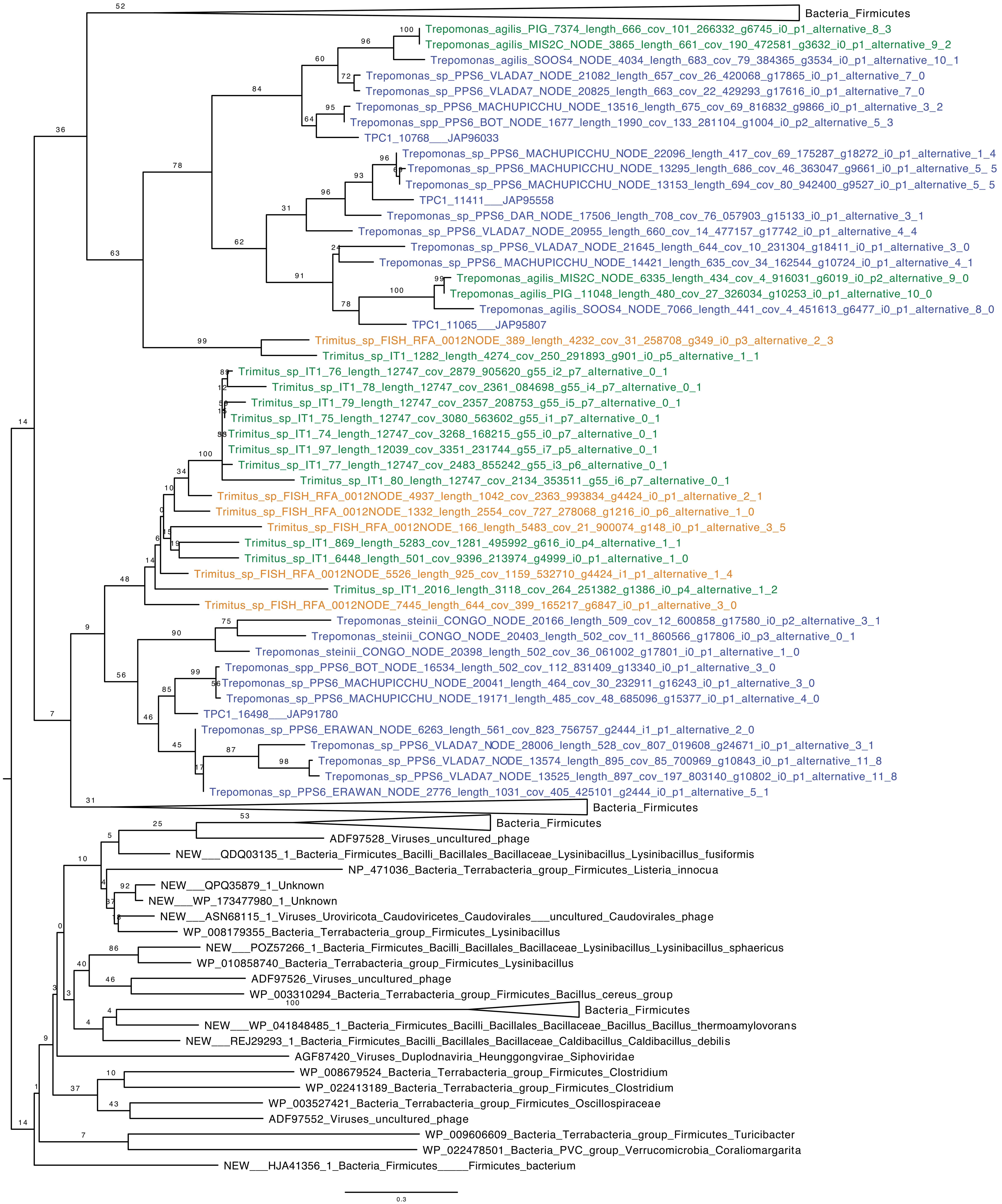

NlpC/P60

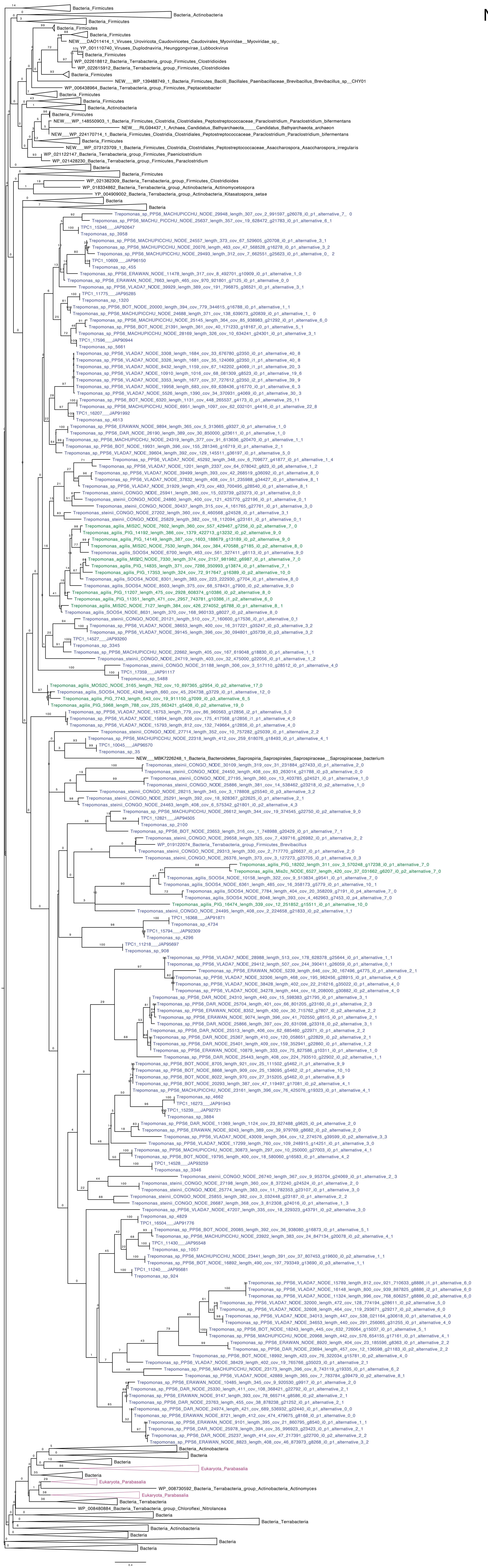

Adenosine deaminase (ADA)

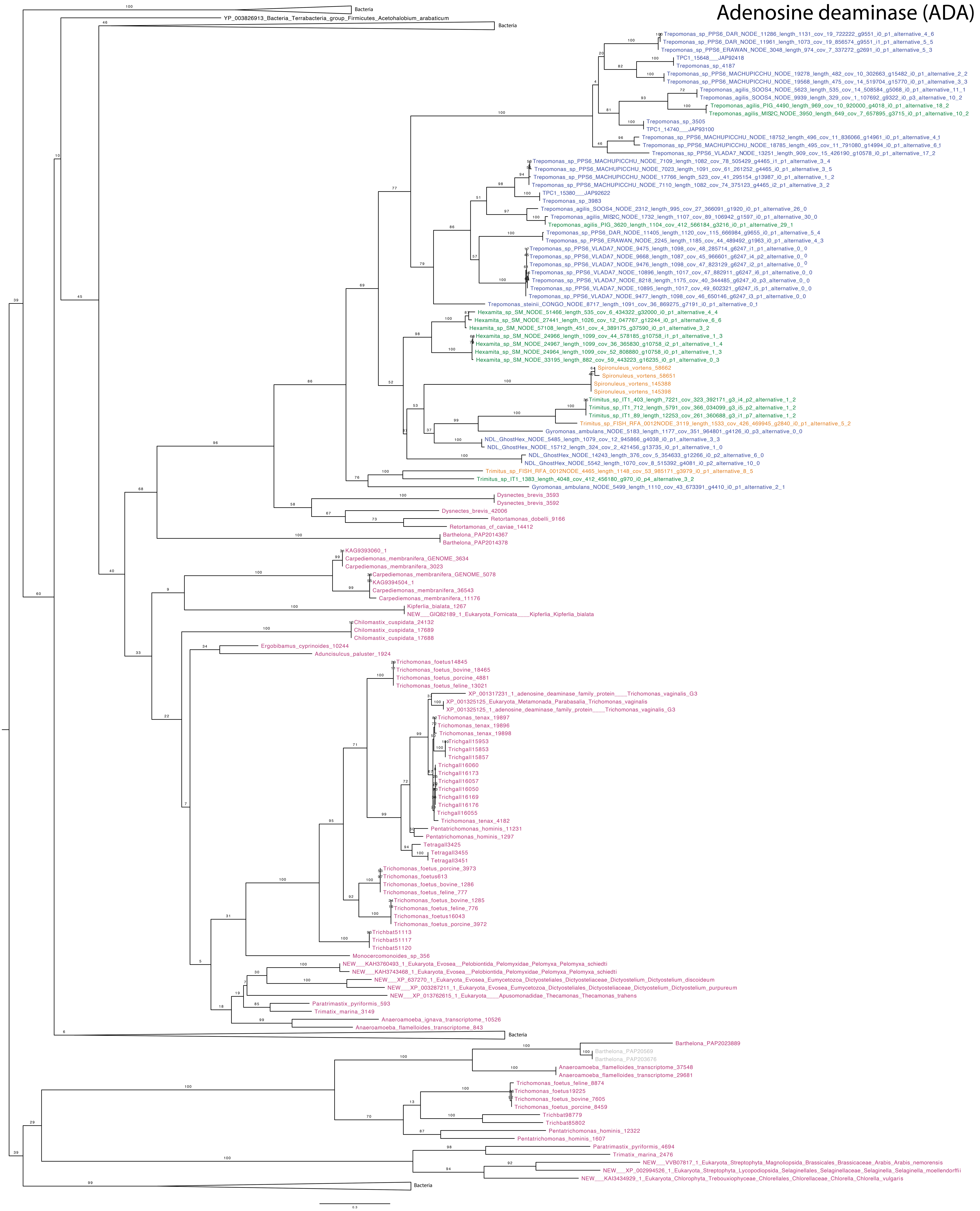

Ribonucleotide reductase (RNR)

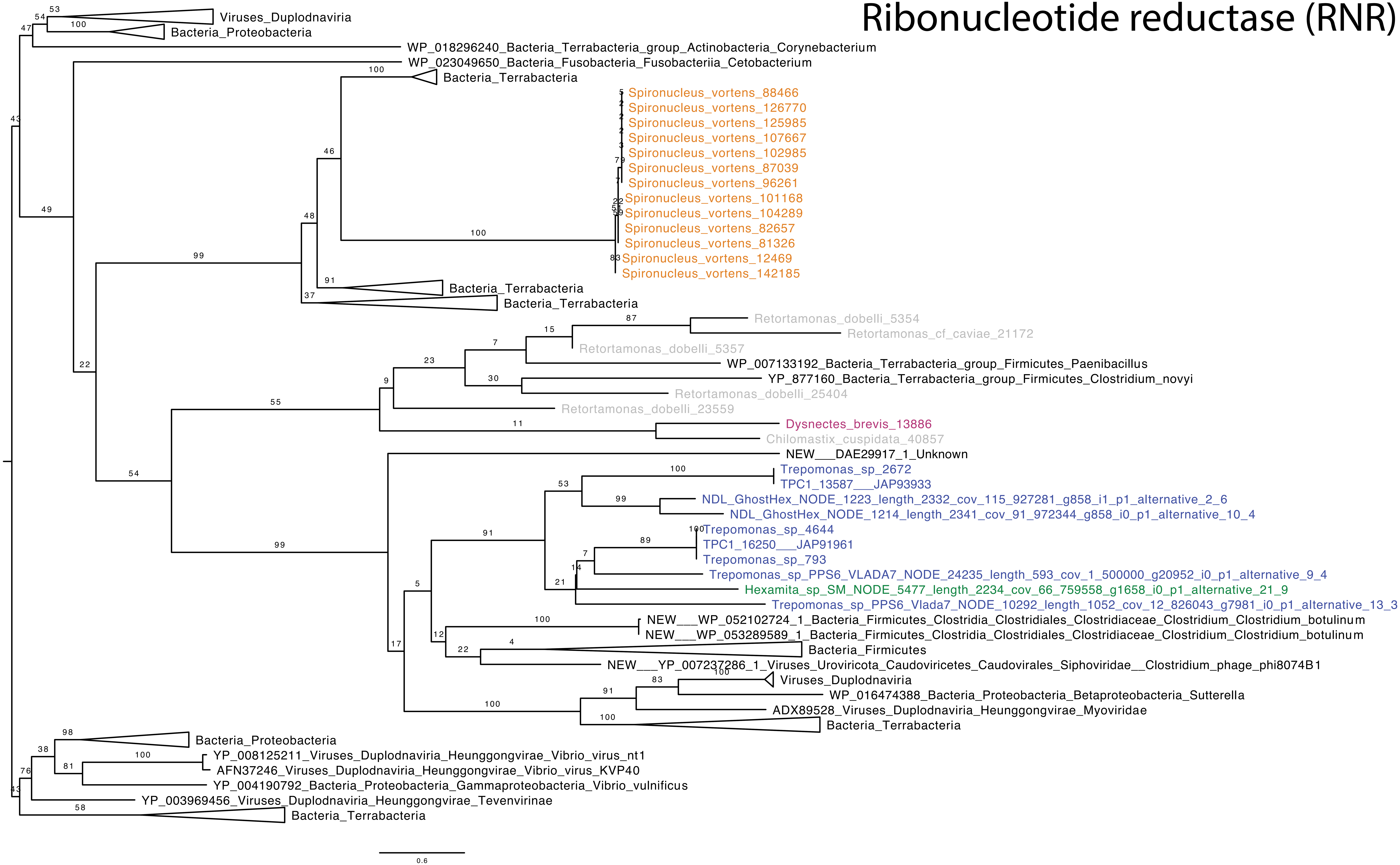

Deoxyuridine triphosphatase (DUT)

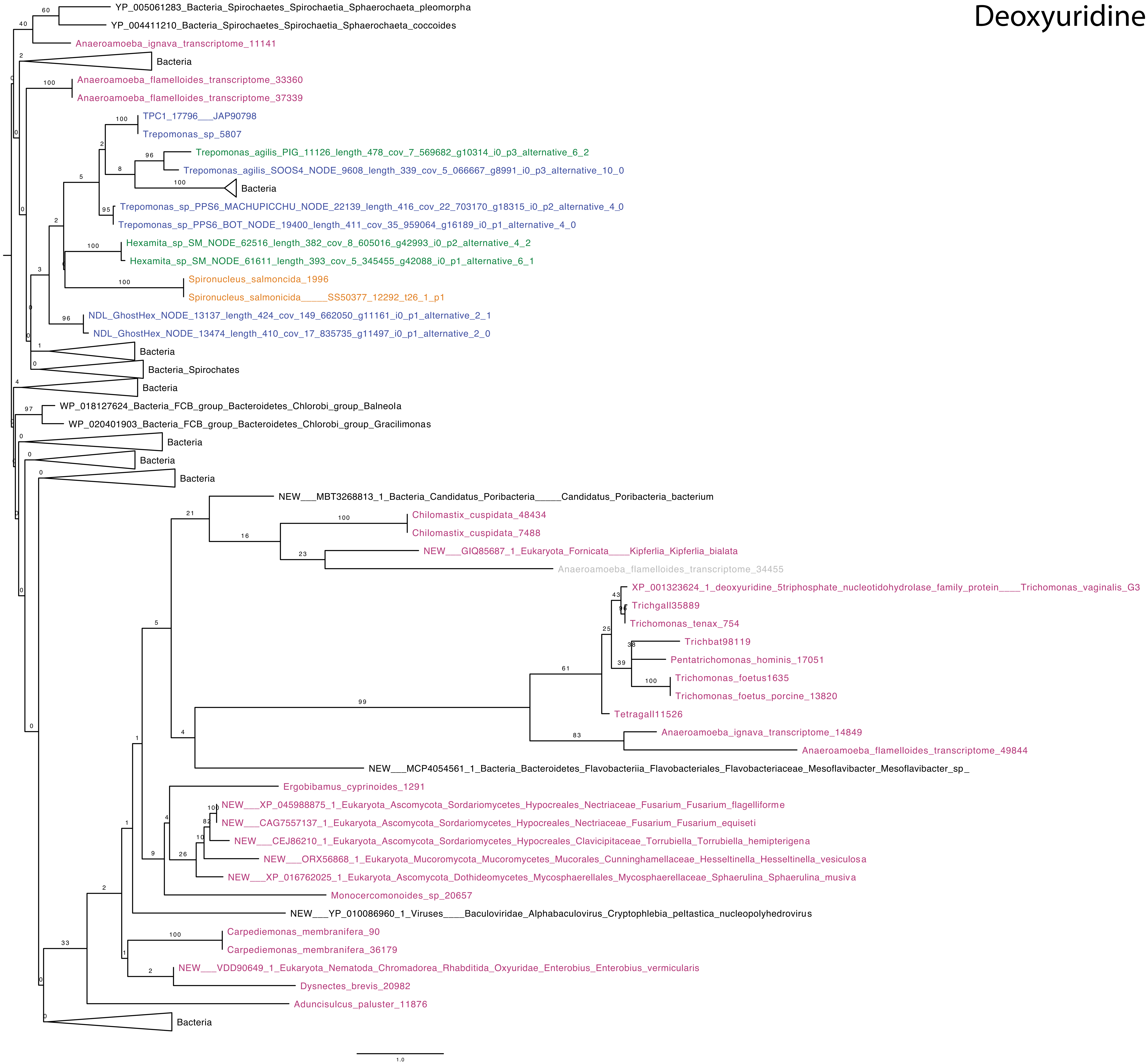

Uracil phosphoribosyltransferase (UPRT)

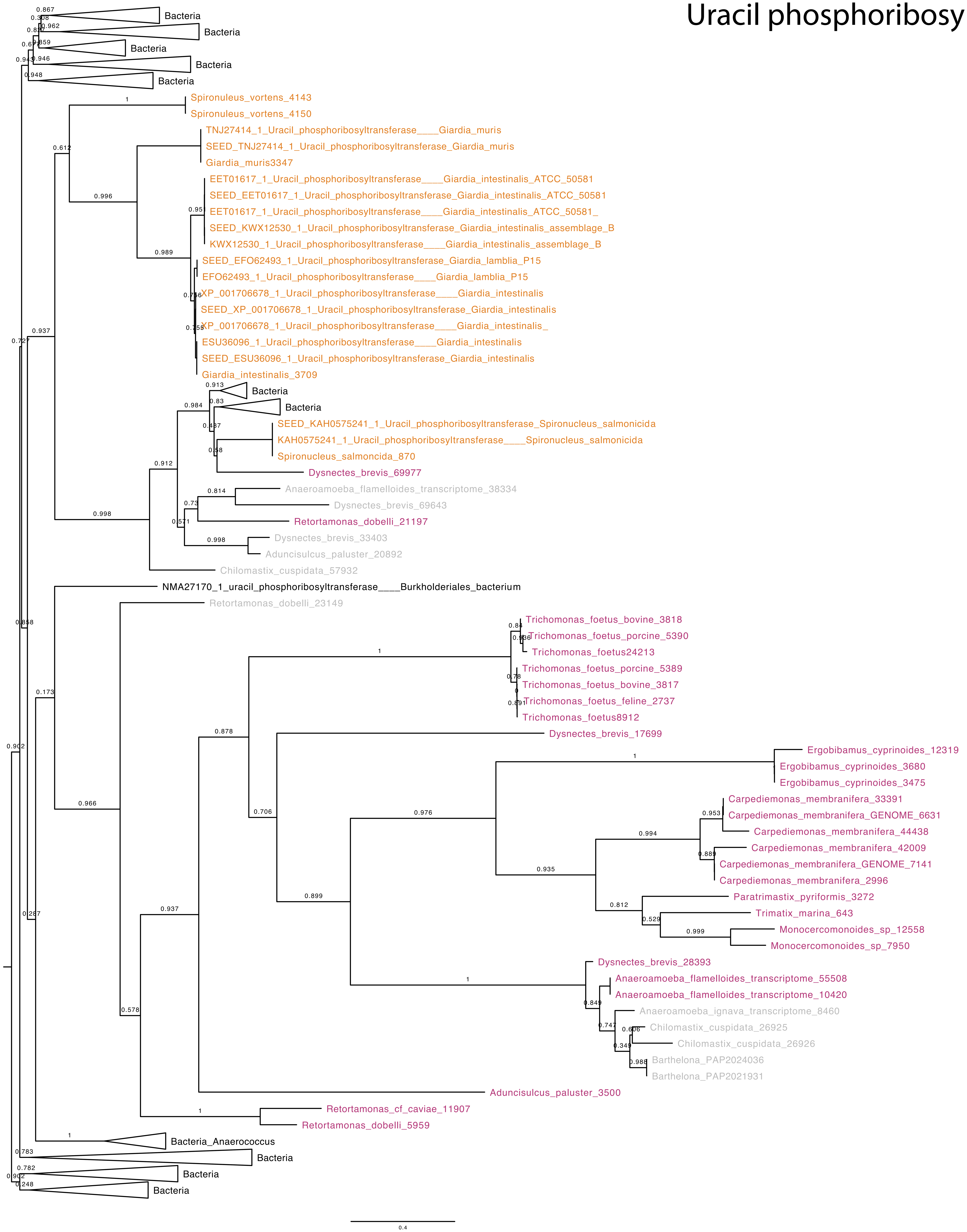

Purine nucleoside phosphotylase (PNP)

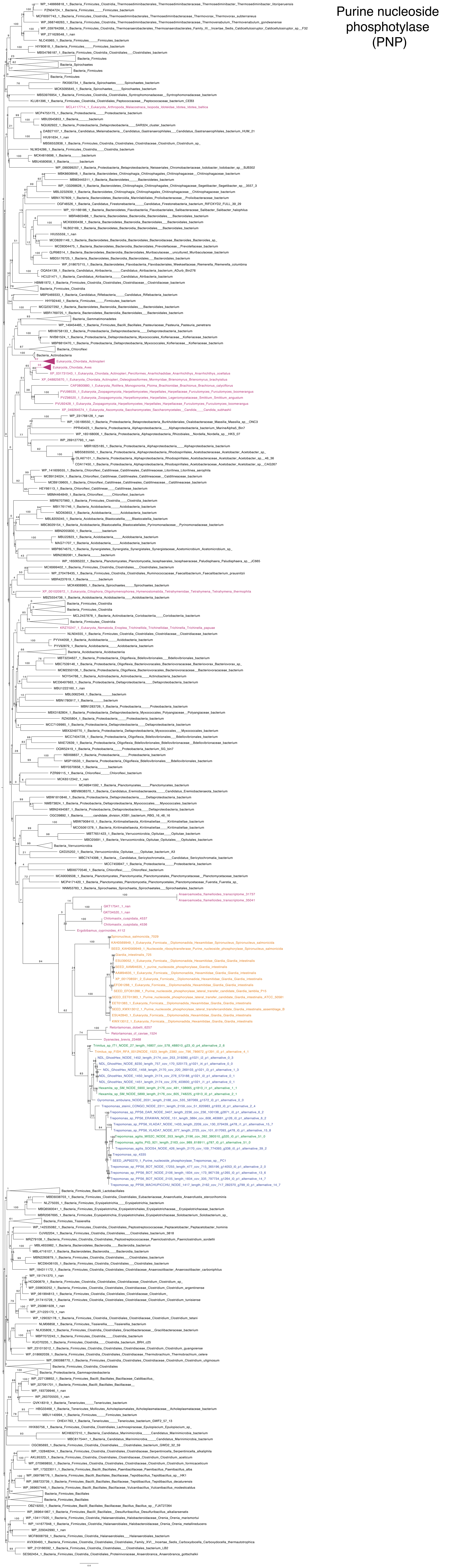

Supplement: Supplementary file 6 — Additional file 6: Fig. S3. Maximum Likelihood trees of the selected HGT datasets. Trees were computed using IQ-TREE with C20+LG+G+F model, statistical support was inferred from 100 rapid bootstrap replicates computed in RAxML with LG4X+G model. Host associated diplomonads are marked in orange, free-living in blue, and green shows diplomonads whose lifestyle is uncertain. Other eukaryotes are colored in red. To improve readability of the trees, large bacterial or eukaryotic clades were collapsed. [file 12915_2024_2013_MOESM6_ESM.pdf]
